# HIV-1 variants are archived throughout infection and persist in the reservoir

**DOI:** 10.1371/journal.ppat.1008378

**Published:** 2020-06-03

**Authors:** Kelsie Brooks, Bradley R. Jones, Dario A. Dilernia, Daniel J. Wilkins, Daniel T. Claiborne, Samantha McInally, Jill Gilmour, William Kilembe, Jeffrey B. Joy, Susan A. Allen, Zabrina L. Brumme, Eric Hunter

**Affiliations:** 1 Emory Vaccine Center, Emory University, Atlanta, Georgia, United States of America; 2 British Columbia Centre for Excellence in HIV/AIDS, Vancouver, British Columbia, Canada; 3 Human Immunology Lab, International AIDS Vaccine Initiative, London, England, United Kingdom; 4 Zambia-Emory HIV Research Project, Lusaka, Zambia; 5 Department of Medicine, University of British Columbia, Vancouver, British Columbia, Canada; 6 Department of Pathology & Laboratory Medicine, Emory University School of Medicine, Atlanta, Georgia, United States of America; 7 Faculty of Health Sciences, Simon Fraser University, Burnaby, British Columbia, Canada; Vaccine Research Center, UNITED STATES

## Abstract

The HIV-1 reservoir consists of latently infected cells that persist despite antiretroviral therapy (ART). Elucidating the proviral genetic composition of the reservoir, particularly in the context of pre-therapy viral diversity, is therefore important to understanding reservoir formation and the persistence of latently infected cells. Here we investigate reservoir proviral variants from 13 Zambian acutely-infected individuals with additional pre-therapy sampling for a unique comparison to the ART-naïve quasispecies. We identified complete transmitted/founder (TF) viruses from seroconversion plasma samples, and additionally amplified and sequenced HIV-1 from plasma obtained one year post-infection and just prior to ART initiation. While the majority of proviral variants in the reservoir were most closely related to viral variants from the latest pre-therapy time point, we also identified reservoir proviral variants dating to or near the time of infection, and to intermediate time points between infection and treatment initiation. Reservoir proviral variants differing by five or fewer nucleotide changes from the TF virus persisted during treatment in five individuals, including proviral variants that exactly matched the TF in two individuals, one of whom had remained ART-naïve for more than six years. Proviral variants during treatment were significantly less divergent from the TF virus than plasma variants present at the last ART-naïve time point. These findings indicate that reservoir proviral variants are archived throughout infection, recapitulating much of the viral diversity that arises throughout untreated HIV-1 infection, and strategies to target and reduce the reservoir must therefore permit for the clearance of proviruses encompassing this extensive diversity.

## Author summary

Despite reducing viremia to levels below the limit of detection in standard assays, effective antiretroviral therapy (ART) does not eradicate cells latently infected with HIV-1. These cells serve as a reservoir for viral rebound if therapy is interrupted; thus, understanding the composition of the reservoir may yield further targets for HIV-1 cure strategies. We have taken a genetic approach to elucidating the reservoir in 13 Zambian subtype C seroconvertors who were followed longitudinally through ART initiation and virologic suppression. In five of the 13 individuals, provirus sequences identical to or differing by five or fewer nucleotides from the transmitted/founder virus were detected, indicating archiving and persistence of early infection variants for more than six years following infection. While the majority of proviral variants in latently infected cells were most closely related to plasma virus circulating immediately prior to treatment initiation, additional variants dating to intermediate time points in the infection were also observed. These findings demonstrate that virus is archived during all stages of ART-naïve infection, and these variants persist throughout ART. HIV-1 cure strategies to eliminate the reservoir must address the broad genetic diversity of a within-host proviral quasispecies including variants archived from acute through chronic infection.

## Introduction

Although over 23 million individuals living with HIV were receiving ART by the end of 2018, only two have been cured following stem-cell transplantation of HIV-1 resistant cells [1–4]. ART alone is not curative due to the persistence and proliferation of latently infected CD4+ T cells harboring intact but quiescent proviruses unaffected by antiretrovirals that target stages of active viral replication [5–11]. This long-lived and potentially self-renewing population of latently infected cells can serve as a reservoir for viral rebound in the event of treatment cessation [12–16], and efforts to understand the reservoir are therefore essential to HIV-1 cure strategies. Genetic approaches investigating the reservoir have sequenced rebounding virus in HIV-1 patients undergoing treatment interruption, reactivated virus from latently infected cells stimulated *in vitro*, and proviral populations during ART [14–26]. These studies provided critical insights into the complex nature of provirus remaining during treatment, only a small fraction of which is capable of replicating, and a further subset of which reactivates with treatment interruption [22]. The sources and establishment of this reservoir are of considerable interest, and although the reservoir is seeded beginning in very early stages of infection [5, 12, 14, 20, 27–29], few investigations have explored the relationship of the reservoir to early infection, pre-therapy viral variants. The extent to which these variants may persist in the reservoir during virologically suppressive ART is incompletely understood.

Recent studies examining associations between transmitted virus, its descendent quasispecies in chronic ART-naïve infection, and the reservoir include genetic analyses of amplified virus by Brodin et al. [30] and Abrahams et al. [31], particularly from quantitative viral outgrowth assay (QVOA) as in Abrahams et al. [31], while additional work from Jones et al. [32] infers age of latent proviral genes in relation to pre-therapy plasma variants. All three groups describe heterogenous populations of proviral sequences that do not indicate ongoing evolution during virologically suppressive ART [30–32]. Furthermore, all three studies observe proviral variants that are inferred to be most closely related to sequences circulating in the plasma immediately prior to the start of treatment as well as variants contemporaneous with the earliest pre-therapy sample [30–32]. The frequencies of proviral variants dating to particular pre-therapy eras are distinct in each study, with Brodin et al. [30] and Abrahams et al. [31] describing 60% and 71%, respectively, of proviral variants during treatment as most closely related to pre-therapy sequences from immediately prior to treatment initiation, while these frequencies are higher than that described in Jones et al. [32]. Given the interpatient variability in proviral population structure present in these studies, it is perhaps unsurprising for discrepancies in findings as well.

In this study, we examined reservoir proviral sequences in the context of pre-therapy plasma HIV-1 RNA diversity and evolution in 13 Zambian seroconvertors. Critically, our reconstruction of within-host HIV-1 evolution includes the inference of the near full-length transmitted/founder virus from single genome amplification, allowing us to investigate the possible long-term persistence of this sequence within the reservoir. Utilizing the phylogenetic approaches developed by Jones et al. [32] and additional analyses to assess the reservoir during short-term ART, our findings indicate that latent proviral diversity broadly reflects plasma HIV-1 RNA diversity during the period of pre-therapy infection. A majority of variants appear most closely related to those circulating in plasma near the time of ART initiation, but the reservoir quasispecies can in some individuals include variants present at the time of transmission, and demonstrates persistence of variants archived throughout ART-naïve infection.

## Results

### Participant selection and sampling methods

**Fig 1:**
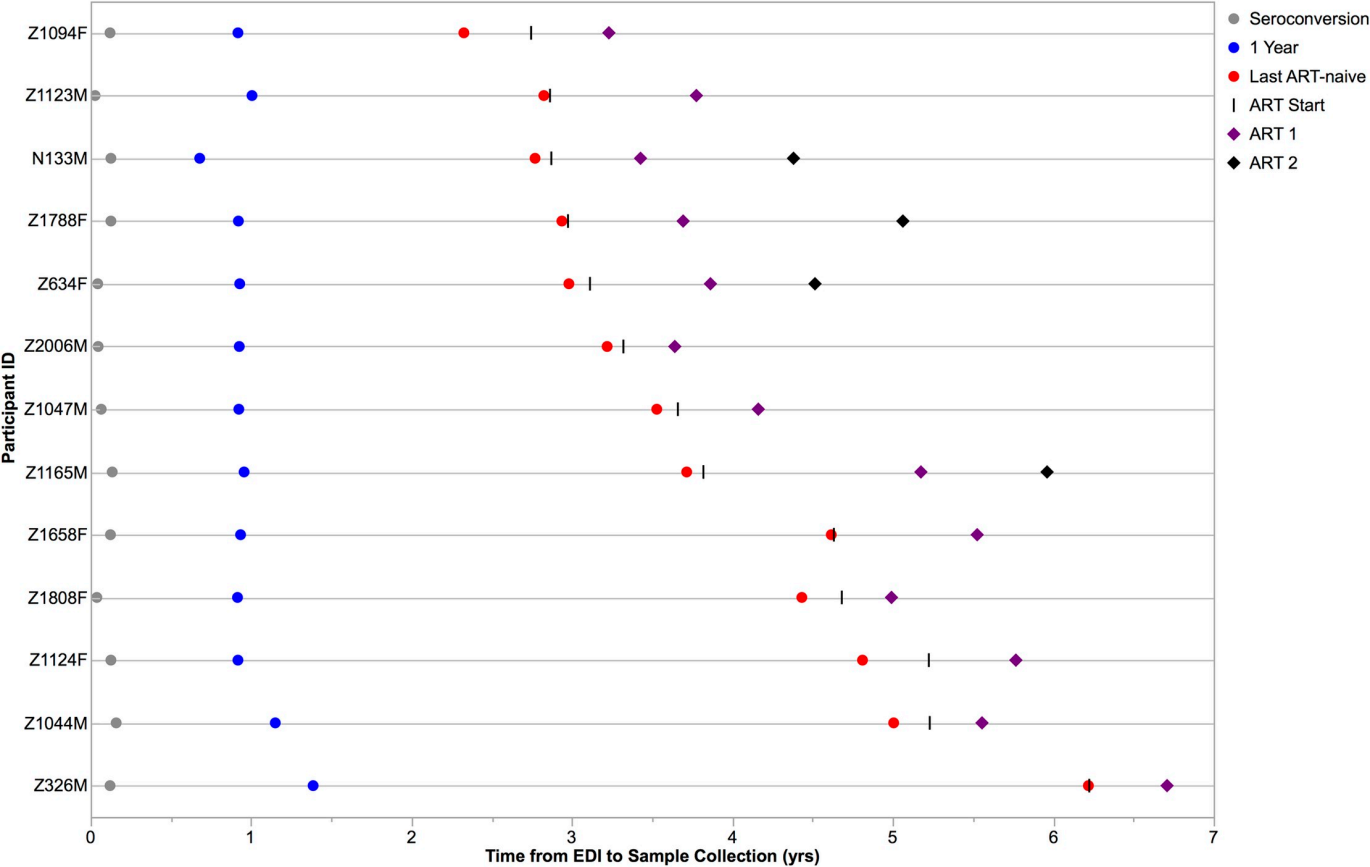
Sampling strategy for study participants. Viral RNA was reverse transcribed, amplified, and sequenced from pre-therapy plasma samples, while proviral DNA was amplified from cells collected during treatment. For six individuals (IDs Z1094F, Z1123M, Z1788F, Z1047M, Z1165M, and Z1658F), we additionally amplified and sequenced proviral DNA from cells at the last pre-therapy time point (red) to assess divergence from the transmitted/founder virus of contemporaneous sequences from plasma vs. cells. EDI = Estimated Date of Infection.

We identified 13 Zambian seroconvertors from the Zambia-Emory HIV Research Project for study according to the following criteria: ART-naïve infection of at least two years, subsequent ART with viremia <50 copies/mL at one or more time points following therapy initiation, and sample availability during treatment (Table 1). All participants received combination ART per country guidelines. We amplified and sequenced a minimum of seven near full-length genomes (NFLGs) by single genome amplification (SGA) from the earliest HIV+ plasma sample available for each participant (seroconversion sample), which was collected a median of 44 days post-estimated date of infection (EDI) (Table 1 and Fig 1). Sequencing was performed using Pacific Biosciences Single Molecule, Real-Time (SMRT) sequencing [33], and the transmitted/founder (TF) virus was inferred as the consensus of the low-diversity NFLGs (S1A Fig). Participant Z1658F was determined to have been infected with two TF viruses (S1 Fig). We additionally utilized SGA and SMRT sequencing to amplify and sequence an approximately 3.6 kb amplicon spanning the *vpu*, *env*, and *nef* genes from plasma samples collected approximately one year following the EDI, as well as from the last pre-therapy time point available (Fig 1). For six individuals, we additionally amplified and sequenced proviral DNA from the last pre-therapy time point to assess the divergence of sequences from the TF virus when collected from the cellular compartment rather than plasma.

**Table 1:** Participant details.

Participant ID	Last HIV- Test Date	First HIV+ Test Date	Estimated Date of Infection (EDI)^a^	EDI to SC Sample (days)	EDI to ART Start (years)	ART Regimen	Time on ART to ART Sample 1, 2 (years)
Z1094F	08-Apr-2008	04-Jul-2008	21-May-2008	44	2.75	EFV, TDF, FTC	0.48
Z1123M	14-Mar-2008	12-Apr-2008	02-Apr-2008	10	2.86	EFV, TDF, FTC	0.91
N133M	16-May-2007	16-Aug-2007	01-Jul-2007	46	2.87	EFV, TDF-FTC	0.56, 1.51
Z1788F	10-Nov-2006	10-Feb-2007	26-Dec-2006	46	2.98	EFV, TDF, FTC	0.72, 2.09
Z634F	22-Jun-2007	24-Jul-2007	08-Jul-2007	16	3.11	EFV, TDF-FTC	0.75, 1.40
Z2006M	09-Apr-2009	13-May-2009	26-Apr-2009	17	3.32	NVP, AZT, 3TC/EFV, TDF, FTC	0.32
Z1047M	24-May-2007	14-Aug-2007	31-Jul-2007	24	3.66	EFV, TDF, FTC	0.50
Z1165M	08-Dec-2005	16-Mar-2006	26-Jan-2006	49	3.82	ABC, 3TC, EFV	1.36, 2.14
Z1658F	11-Mar-2006	08-Jun-2006	24-Apr-2006	45	4.63	ABC, 3TC, NVP	0.89
Z1808F	18-Dec-2007	24-Jan-2008	10-Jan-2008	14	4.68	EFV, TDF, FTC	0.31
Z1124F	15-Feb-2006	18-May-2006	02-Apr-2006	46	5.22	ABC, 3TC, NVP	0.54
Z1044M	29-Nov-2005	25-Mar-2006	26-Jan-2006	58	5.23	AZT, 3TC, EFV	0.33
Z326M	21-Nov-2006	17-Feb-2007	04-Jan-2007	44	6.22	EFV, TDF, FTC	0.48
**MEDIAN**	**NA**	**NA**	**NA**	**44**	**3.66**	**NA**	**0.54, 1.80**

N = Ndola, Zambia; Z = Lusaka, Zambia; M = Male; F = Female; SC = seroconversion

^a^See materials and methods for calculation of EDI

For all participants, the *vpu*-*env*-*nef* amplicon was also generated by SGA from DNA of peripheral white blood cells collected during treatment; for four individuals, proviral sequences were also sampled at a second, later time point during ART (Table 1 and Fig 1). All sequences analyzed across time points were free of obvious defects such as nonsense mutations, INDELs resulting in disruption of an open reading frame, or in-frame INDELs of more than 90 nucleotides (up to 90 accepted to accommodate Env variable loops). In total, 1,275 sequences, excluding APOBEC hypermutants, were generated and analyzed for all participants and time points (S1 Table).

### Infection date estimates

**Fig 2:**
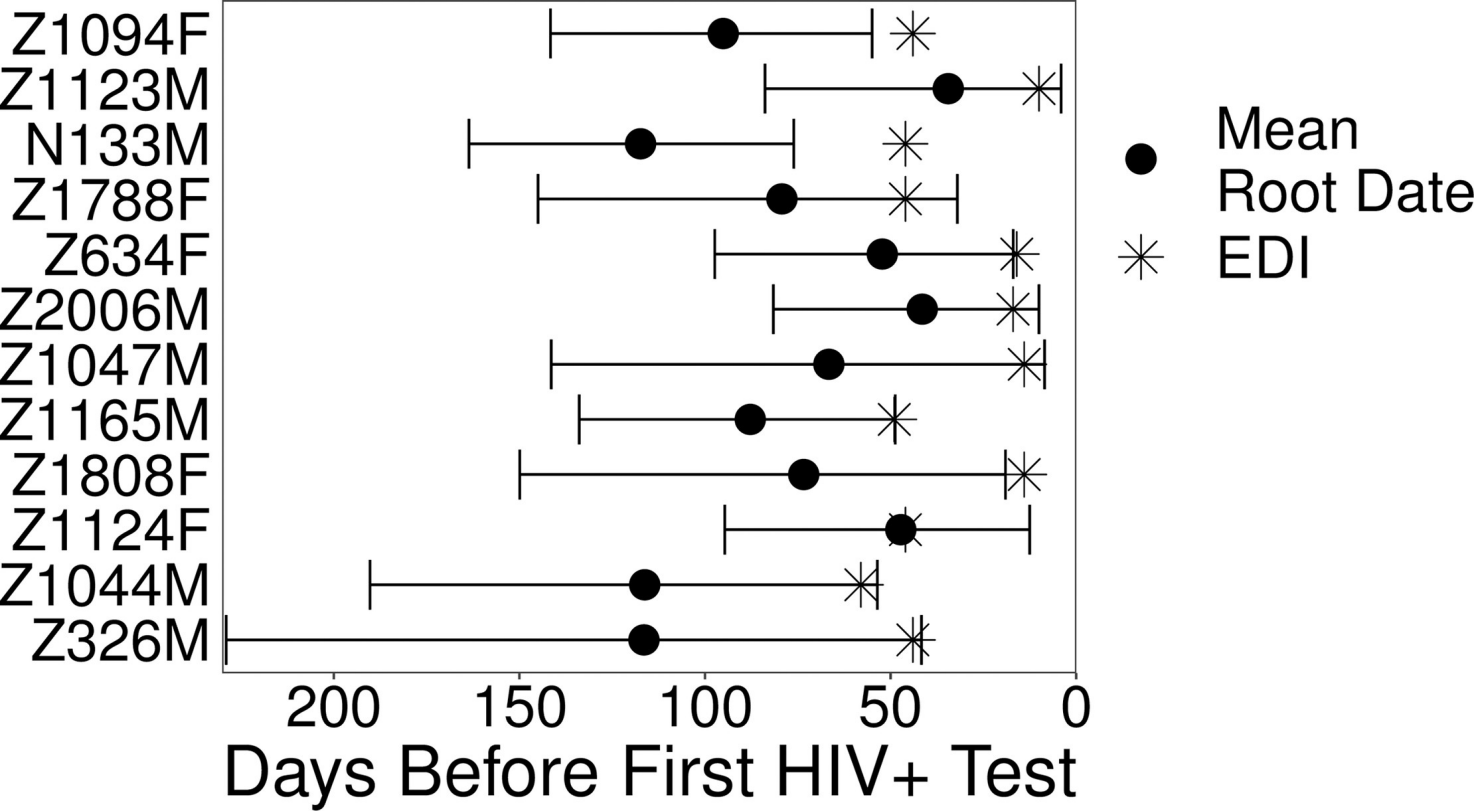
Bayesian root date estimation. Bayesian inference was used to estimate the root date, or time of infection, from pre-therapy plasma *env* sequences. The 95% highest posterior density intervals surround the mean estimated root dates of the Bayesian inference (circles). The clinically estimated infection dates (EDIs) are depicted as stars.

Although participants studied in this investigation were routinely tested for HIV, providing narrow windows for infection between the last HIV negative and first positive tests, estimation of the infection date within these time frames is calculated as the midpoint of the antibody test dates. To corroborate our clinically-based EDIs from HIV testing in the single-variant infections, we used Bayesian approaches to infer the mean root date, a proxy for infection date, directly from sequence data. We used all pre-therapy *env* sequences without evidence of hypermutation or recombination for the analysis, annotated with the sample collection dates. Though Bayesian-estimated mean root dates predated the clinical EDI in all cases, the 95% confidence intervals of the Bayesian root dates overlapped the clinical EDIs in all but four infections (Fig 2). These results confirm the early nature of the infections when sampled at the seroconversion time point.

### Reservoir variants are distributed throughout the phylogenetic trees of viral sequences from each individual

**Fig 3:**
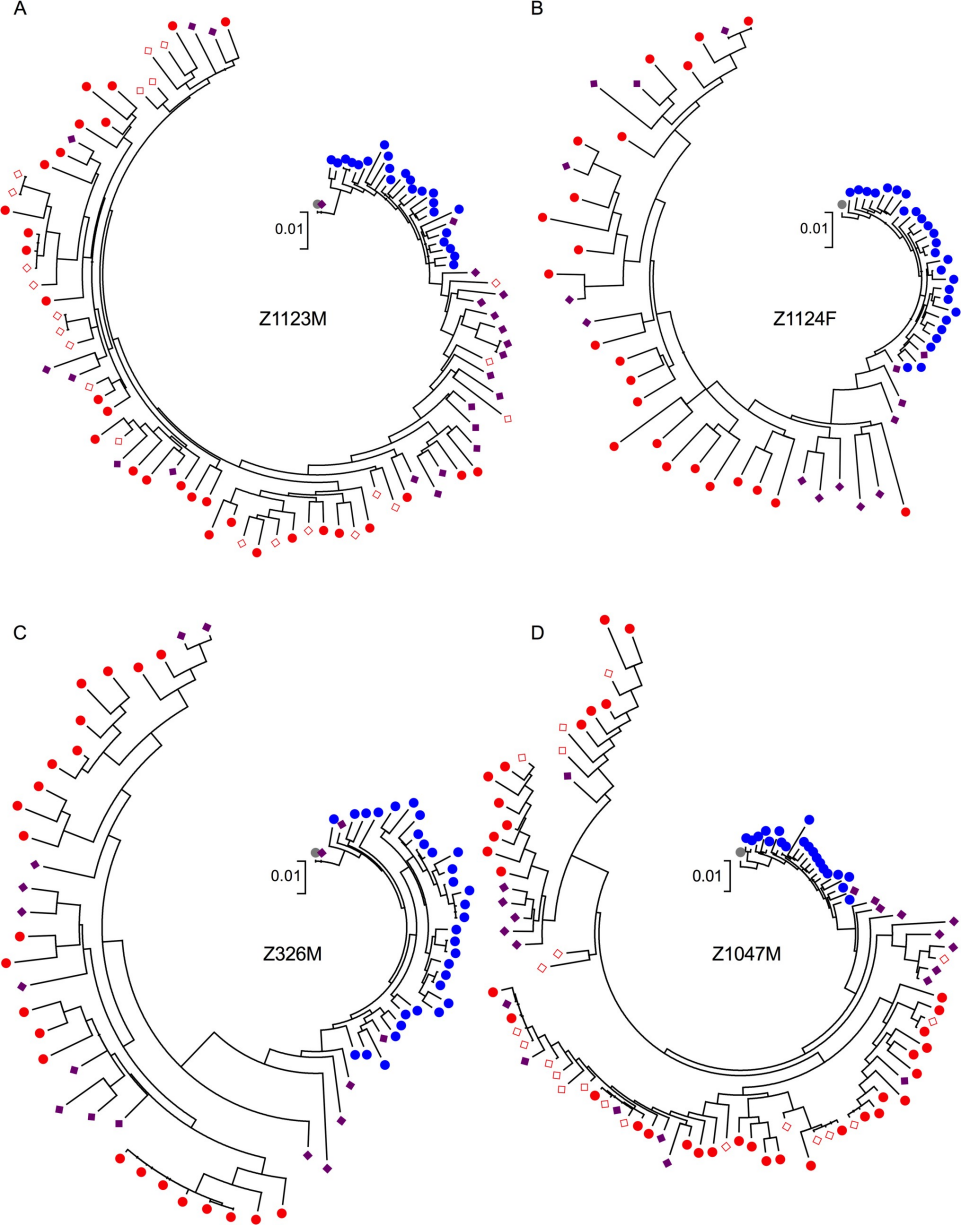
Maximum-likelihood (ML) trees for viral and proviral variants in four individuals. Representative ML phylogenetic trees for participants Z1123M (A), Z1124F (B), Z326M (C), and Z1047M (D) rooted on the respective transmitted/founder (TF) virus (grey) identified from the seroconversion sample and depicting all viral variants from one year post-infection (blue), the last ART-naïve sample (red), and during treatment (purple diamonds). Variants from cells collected at the last ART-naïve time point are shown in open red diamonds, while all plasma variants are in filled circles.

**Fig 4:**
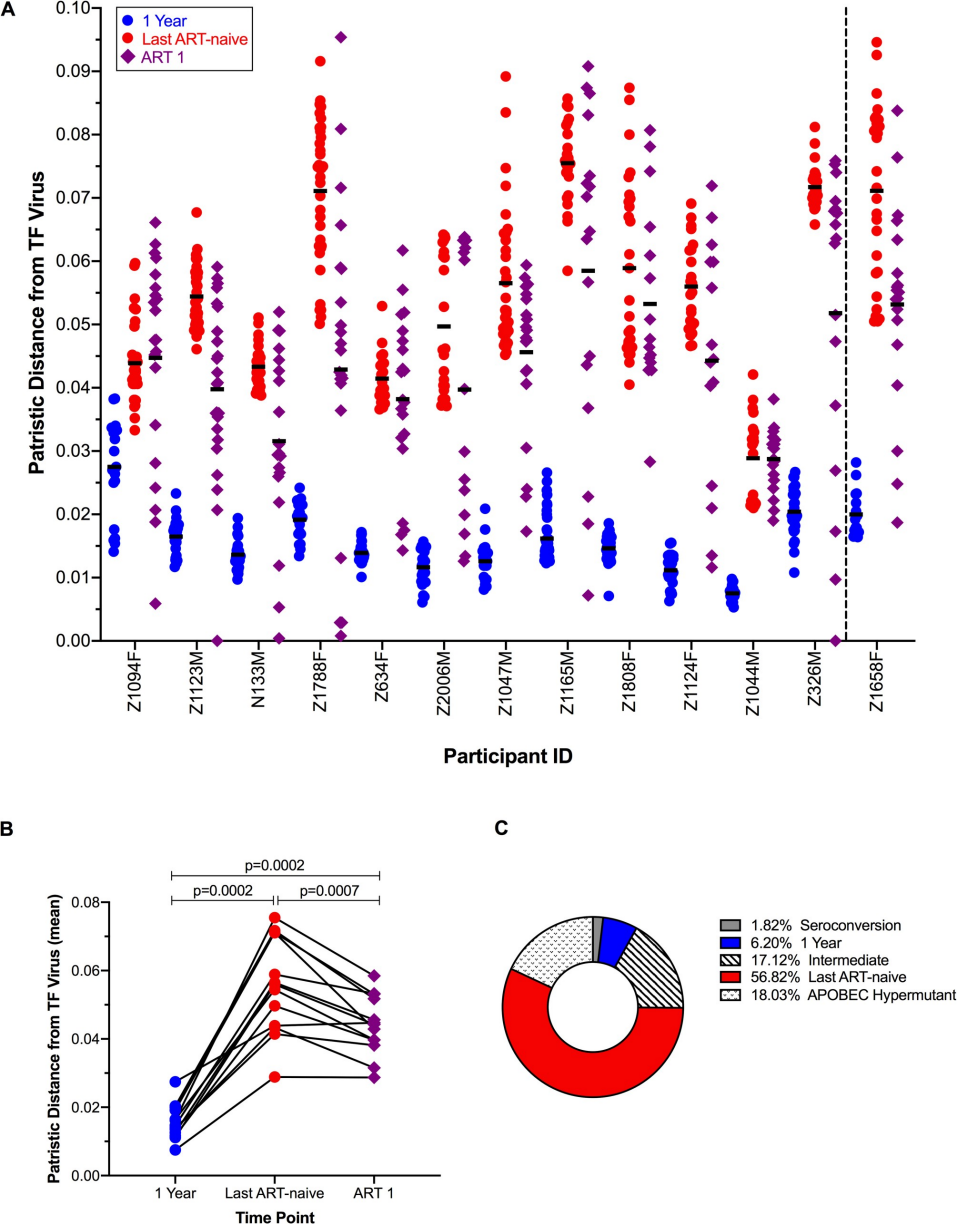
Divergence from transmitted/founder (TF) virus increases during ART-naïve infection and decreases on treatment. (A) Patristic distances from the TF virus, or root-to-tip values, from the maximum-likelihood (ML) trees are shown for all 13 participants, with the single instance of multivariant infection in participant Z1658F shown at the far right. Where two samples were assessed during treatment, only the first is shown here. Mean values are indicated by horizontal black bars. (B) Summary of the mean intrapatient patristic distances, where the mean distance is significantly different between each time point assessed (Wilcoxon matched-pairs signed rank tests). (C) Proportion of proviruses seeded into the reservoir, by era, as estimated from the placement of reservoir sequence in the phylogeny. The mean values of proportions for all 13 volunteers are shown, while proportions for each participant are given in S4 Fig. A majority of variants are most closely related to variants present at the last ART-naïve time point. The percentage of variants demonstrating APOBEC hypermutation is also indicated, though these sequences were excluded from analysis.

For all 13 participants, a maximum-likelihood phylogeny was inferred from within-host alignments of plasma RNA and proviral DNA *vpu-env*-*nef* sequences from all time points, where the tree was rooted on the inferred TF virus (Fig 3 and S2 Fig). This permits insight into the divergence of all descendant variants in each population from the TF virus, as well as the inferred evolutionary relationships between them. Divergence of plasma sequences from the TF virus by patristic (root-to-tip) distances increases significantly from one year post-infection to the last ART-naïve time point, yet there is a significant decrease in the patristic distances of proviral sequences sampled during treatment compared to those sampled at the last ART-naïve time point (Fig 4B). This decrease does not appear to be an artifact of analyzing sequences from cells versus plasma alone, as ART sequences are significantly closer to the TF virus than sequences from cells at the last ART-naïve time point (S3 Fig). Sequences with minimal divergence from the TF virus are persisting in the reservoir, as can be seen for participants Z1123M and Z326M (Fig 3A and 3C), where sequences identical to the TF were recovered in the reservoir, having persisted for over two years and six years of ART-naïve infection, respectively.

Although proviral variants closely related to and including the TF virus persist in the reservoir, reservoir variants are distributed throughout the viral phylogenies for each individual. Plasma variants from one year post-EDI and the last pre-therapy time point exhibited a "ladder-like" topology characteristic of within-host phylogenies [34], where plasma sequences from a given time point formed distinct clades in all intrapatient trees, and reservoir variants fell within or between these clades. In an initial analysis, we classified individual reservoir variants as being most closely related to variants of the clade within which they fall, or as intermediate in cases where they do not fall within the one-year or last ART-naïve clades (Fig 4C, S4 Fig). Additionally, variants that did not fall within either the one year post-infection or last ART-naïve clade and were within six nucleotide changes from the TF virus were classified as seroconversion variants, as they exhibit divergence from the TF virus equivalent to the level of diversity among sequences from the serconversion time point. In 10 of 13 individuals, we observed at least one proviral variant classified as most closely related to seroconversion or one year post-EDI plasma variants, while in all participants we observed proviral variants most closely related to the last ART-naïve plasma variants, with these sequences making up the greatest portion of the total proviral populations overall (Fig 4C). Taken together, we consider the presence of TF or seroconversion variants, as well as variants classified as intermediate and chronic, to indicate archiving of viral sequences throughout infection.

### Dating of provirus integration indicates variant archiving throughout infection

**Fig 5:**
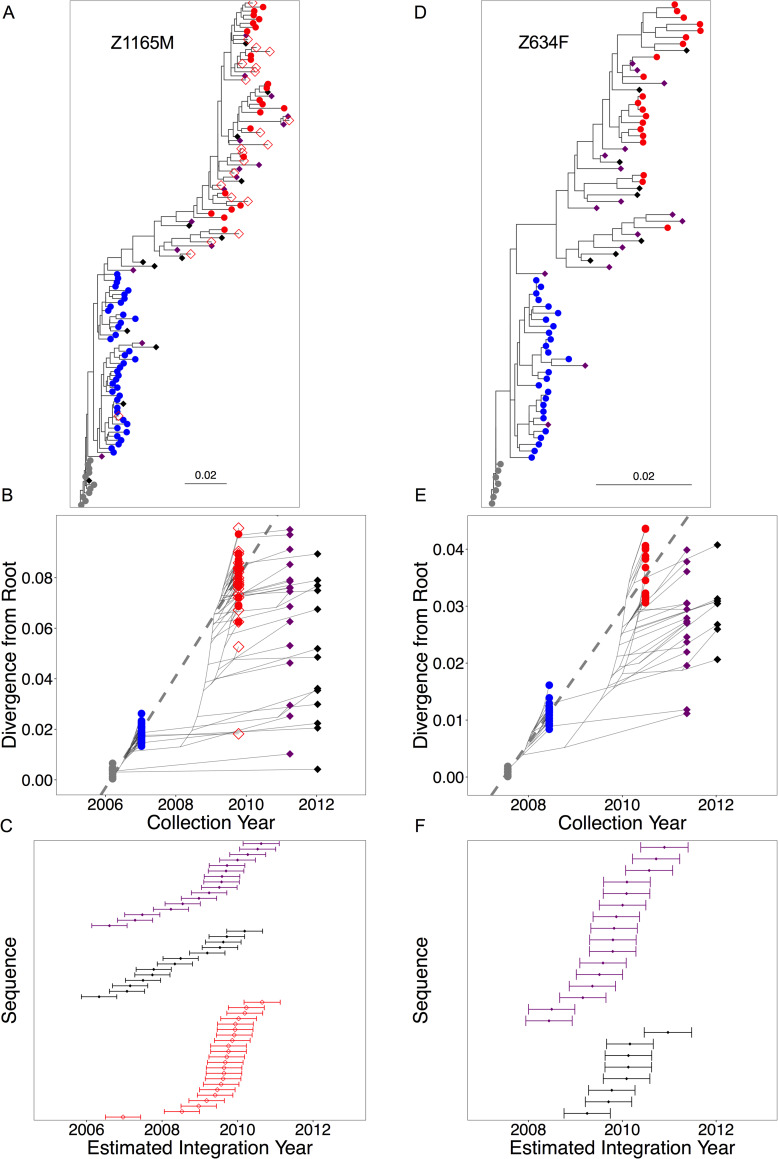
Regression-based inference of time of provirus integration. Representative figures for participant Z1165M (A-C) and participant Z634F (D-F). Maximum-likelihood trees of the *env* gene for pre-therapy variants (circles), including individual seroconversion variants (grey), one year sequences (blue), last pre-therapy sequences (red circles, plasma and open red diamonds, cells), and proviral variants (filled diamonds) in A and D. Two samples during treatment were assessed for both participants, with the first in purple, and subsequent in black. Trees were rooted to optimize the correlation between root-to-tip distance and sampling time for all pre-therapy plasma variants. The linear model relating root-to-tip distances to sampling time is shown in the dashed lines of figures B and E, with the pre-therapy variants denoted as colored dots, and the phylogenetic relationships between them denoted as faint grey lines. Proviral variants from samples collected during treatment are shown in filled diamonds in the same manner. The estimated integration dates of the proviral variants and 95% confidence intervals are shown in the plots C and F. Figures for additional individuals are in S5 Fig.

While visualization of within-host phylogenies inferred from pre-therapy plasma sequences and proviruses persisting during ART allowed us to estimate the era in which the latter integrated into the reservoir, to more precisely estimate the age of proviral variants with respect to the ART-naïve infection, we applied the method developed by Jones et al. [32]. This method utilizes pre-therapy plasma sequences to develop a model of within-host evolution relative to sampling time, and places reservoir variants at a distinct date along the infection history, rather than into a broad category based on relatedness to discrete pre-therapy sampling. For this analysis, we inferred maximum-likelihood phylogenies from pre-therapy plasma and reservoir proviral *env* sequences, where the root was placed at the location that maximized the correlation between the divergence from the root and sample collection date of the pre-therapy plasma sequences (Fig 5A and 5D, S5 Fig). The pre-ART plasma variants were then used to train a linear model that related their divergence from the root and their sample collection dates. The linear model was used to infer the integration date and 95% confidence interval of the age of each proviral variant based upon its divergence from the root (Fig 5B, 5C, 5E and 5F, S5 Fig).

Consistent with reservoir proviral variants being seeded at various times spanning infection to treatment initiation, there is considerable discrepancy between sample collection dates and inferred integration dates for proviral sequences sampled during ART, with some variants estimated to have been integrated near the time of seroconversion. In a representative case, the point estimates of integration dates for Z1165M indicate a variant was archived within three months of the root date of Feb 21, 2006. In contrast, several variants displaying considerably higher divergence from the root were present as well, including those with estimated integration dates consistent with the last ART-naïve plasma variant date estimates. In participant Z634F, there are two variants dating to approximately one year post-infection, but no earlier variants, and only provirus dating to shortly prior to the initiation of therapy was detected in participants Z1044M and Z1808F (S5 Fig). Interestingly, where cells were sampled at the last ART-naïve time point, such as for participant Z1165M, the integration date estimates for provirus of these cells typically fell slightly after that of the proviral variants persisting during treatment. Across the group of participants, however, and consistent with our initial analysis in Fig 4C, integration date estimates for proviral sequences supported periodic seeding of variants in the reservoir throughout the infection.

### Repeated sampling during ART and persistence of early infection variants

**Fig 6:**
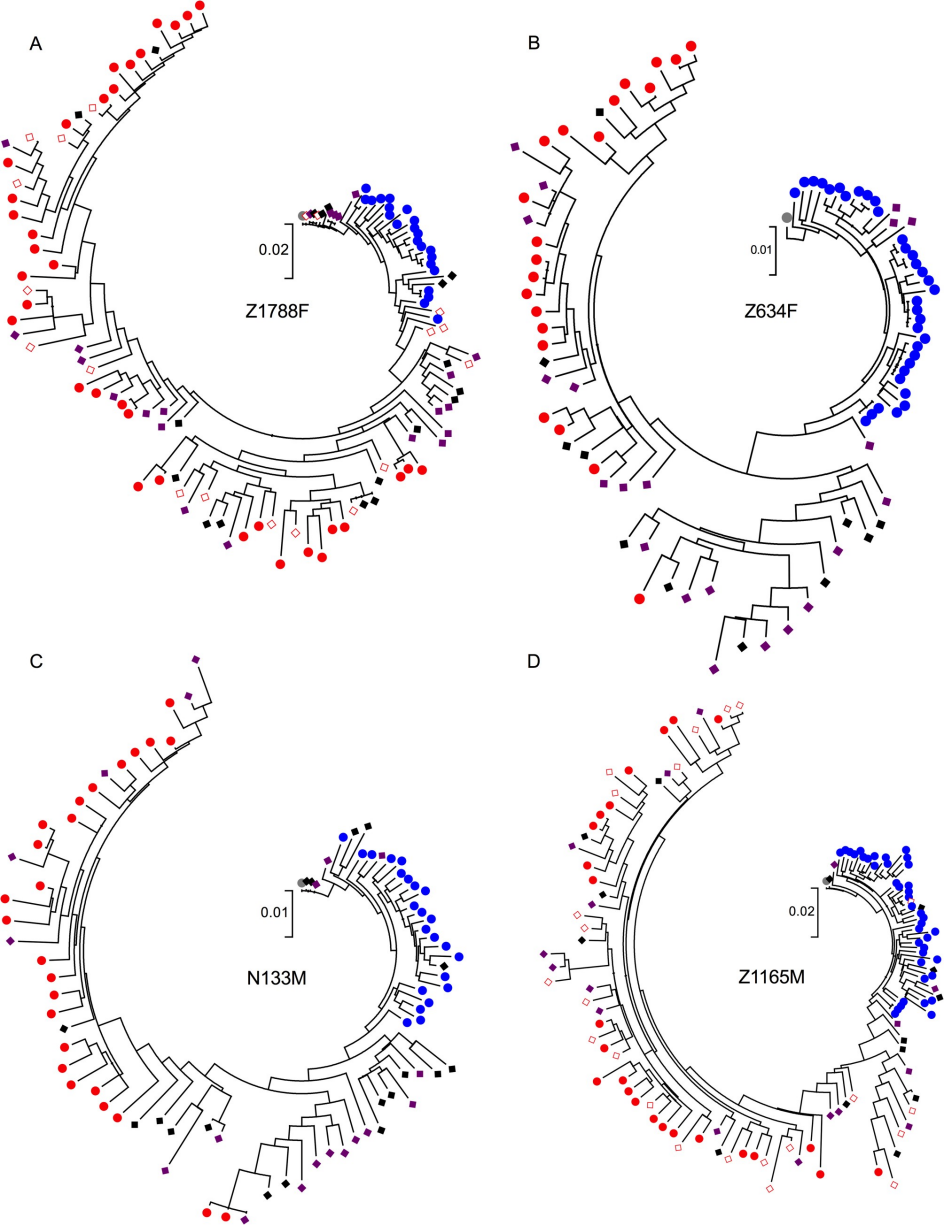
Maximum-likelihood (ML) trees of all variants for participants with two samples during treatment. ML phylogenetic trees for all four participants: Z1788F (A), Z634F (B), N133M (C), and Z1165M (D) rooted on the respective transmitted/founder virus (grey) and depicting all viral variants from one year post-infection (blue), the last ART-naïve sample (red), and during treatment (purple and black diamonds, with second sample in black). Sequences from cells collected at the last ART-naïve time point are in open red diamonds, while plasma sequences are depicted in circles.

The dynamics of proviral decay during short-term ART influence the results of this investigation, as proviral DNA decays most rapidly within approximately the first one to two years of therapy [35–38]. By sampling during this time frame, we therefore may be sampling provirus that persists only transiently rather than comprising the more stable population of latently infected cells with a slower decay rate. To determine if within-host proviral composition was influenced by the relatively short time on treatment, we sampled an additional time point six months to a year later in four participants. All proviral variants without APOBEC hypermutation were included in phylogenetic trees along with all pre-therapy sequences, and phylogenies were again rooted on the TF virus (Fig 6). Early variants were observed throughout the repeated sampling during ART, as proviral variants classified as seroconversion variants were in both the first and second time point during treatment for participant N133M (Fig 6C). For participant Z1165M, a single proviral sequence most closely related to seroconversion sequences was observed in the second time point during treatment (Fig 6D), and in participant Z1788F, seroconversion and early infection variants differing from the TF virus by up to approximately 30 nucleotides were found during the first and second time points following treatment (Fig 6A). Overall, however, proviral sequences from both time points during treatment were intermingled with each other and the sequences sampled prior to treatment.

**Fig 7:**
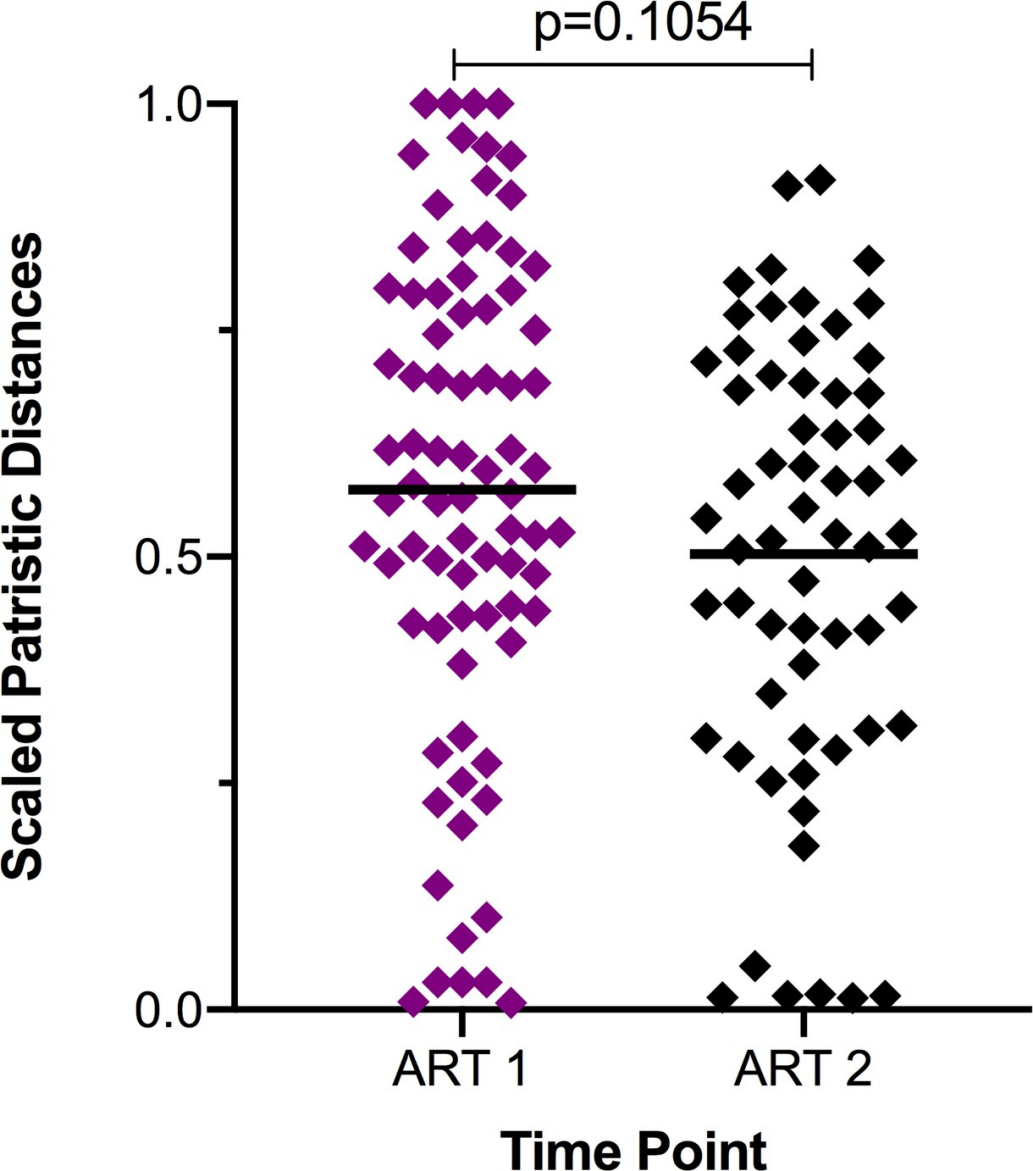
Distance from transmitted/founder (TF) virus decreases with subsequent sampling during treatment. Patristic distances from the TF virus, or root, for reservoir variants as a proportion of the greatest intrapatient patristic distance (tree height) from the maximum-likelihood phylogenetic tree; means shown in horizontal black bars. Distances are lower for variants sampled at the second time point during treatment compared to the first (unpaired t test).

To extend this analysis, we formally compared proviruses sampled at both time points during ART with respect to their genetic divergence from the TF virus. To facilitate combining data across participants, root-to-tip or patristic distances of each reservoir sequence were normalized to the participant's total tree height. Comparison of these scaled root-to-tip distances by sampling time point during ART revealed shorter mean distances for the later reservoir samples compared to the earlier ones (unpaired t test, p = 0.1054, Fig 7). This suggests that, with ongoing treatment, viral variants are not continuing to evolve, as this would bring about an increase in patristic distance. Furthermore, it suggests that early viral variants may be enriched in the reservoir during ART.

## Discussion

We observed that proviral sequences from 13 individuals who had undergone short-term ART were distributed among pre-therapy sequences in phylogenetic trees, with the majority of proviral sequences most closely related to variants from the last ART-naïve time point. However, as analysis of the estimated time of integration for proviral sequences indicates, there is archiving of variants throughout ART-naïve infection, from the earliest time of infection to treatment initiation. This finding is consistent with previous work by Jones et al. [32], but extends the stages of pre-therapy infection explored to acute infection. We identified TF viruses from acute infections with longitudinal follow-up through chronic ART-naïve infection and treatment initiation, while the pre-therapy samples of the two HIV-1 infections investigated in Jones et al. are from chronic infections [32]. Archiving of variants throughout ART-naïve infection is complementary to the observations that the reservoir is smaller and less diverse in individuals beginning treatment early in infection versus during chronic infection [37, 39, 40], since preventing replication with ART ensures a halt in viral evolution and concomitant latent infection with progressively more diverse variants.

Within the diverse populations of proviral sequences we observed, we identified variants that were identical to or contemporaneous with the TF virus after as many as six years of ART-naïve infection and following six to 24 months of ART. These very early, TF-related sequences were observed in five of the 13 individuals sampled and represented from 2.6–7.5% of all reservoir variants in those individuals. It is clear that these very early viral sequences can persist for several years in the absence of therapy, consistent with their integration in long-lived CD4+ T cells. Persistence of ancestral variants is not unprecedented, as several studies assessing drug resistance in patients receiving virologically suppressive ART after a history of non-suppressive therapy found that both ancestral, drug susceptible virus and variants with resistance mutations persist during years of effective treatment [41–44].

Recent studies have shown that a majority of proviruses persisting during ART exhibit large internal deletions or other defects, such as nonsense mutations resulting from APOBEC-induced hypermutation, which render the provirus defective [22, 24]. Due to sample limitations, we assessed approximately one-third of the genome encompassing the *vpu*, *env*, and *nef* genes, and thus cannot exclude the possibility that sequences we have observed as exact matches to the TF virus in this amplicon might contain differences elsewhere in the genome, including mutations and/or deletions that would prevent viral replication. Nevertheless, all of the sequences used for analysis do represent biologically functional gene regions, since sequences with frameshifting INDELs or nonsense mutations were excluded. Unlike Abrahams et al. [31], who used QVOA to characterize sequences reactivated *in vitro*, we are not exclusively addressing the replication-competent reservoir. However, QVOA are known to underestimate the size of the reservoir, as the bulk of replication-competent proviruses are not induced with single or successive rounds of stimulation [22]. Phylogenetic assessment of HIV-1 DNA during virologically suppressive ART serves to address the broad population of persistent provirus within which the replication-competent reservoir is contained, and address its relationship to pre-therapy virus.

In addition to containing TF virus or very early infection variants in some individuals, reservoir proviral populations were overall less evolved from the TF virus than the sequences at the last ART-naïve time point (Fig 4). This finding may be influenced by the short duration of treatment, as all individuals studied here received ART for less than three years at the time of sample collection during treatment, and three participants were sampled within six months of treatment initiation while the reservoir is less stable. However, we did find that early infection variants persisted with continued time on treatment in individuals sampled twice while receiving ART. Furthermore, sequential sampling indicated that with continued time on treatment, the distance of reservoir variants from the TF virus decreased (Fig 7), indicating a potential enrichment for variants dating to earlier in the course of the infection. As viremia rapidly declines in the first phase of viral decay following treatment initiation, followed by a second, slower decay phase [45], latently infected cells decay in stages [35–38], perhaps with those infected most recently by variants circulating in the plasma just prior to treatment initiation decaying first. This mechanism would be consistent with the observation that CD4+ central memory T cells from four to eight years of ART harbor HIV-1 DNA more closely related to early infection sequences than HIV-1 DNA of shorter-lived CD4+ effector memory T cells, in which there is a more prominent decline of HIV-1 DNA with continued time on treatment [23]. However, the relationship between CD4+ differentiation status and the ages of proviruses persisting during ART is by no means clear [46]. Further studies must address the phylogenetic influence of latently-infected cell decay.

As HIV-1 prevention and treatment efforts are scaled up globally, research efforts to reduce and/or eliminate the reservoir in pursuit of an HIV-1 cure are expanding as well. Towards this goal, it is critical to characterize the genetic diversity of the reservoir to assess the variants that HIV-1 eradication strategies must target. Our findings indicate that virus is archived throughout infection, and cure strategies should therefore address the genetic diversity of reservoir proviral quasispecies with many unique variants, including those dating back to the time of infection.

## Materials and methods

### Human subjects

Zambian volunteers were enrolled as heterosexual couples in Couples Voluntary Counseling and Testing (CVCT), with HIV testing and counseling of both partners conducted upon enrollment. Follow-up HIV testing was conducted approximately every three months for the negative partners of serodiscordant couples, and blood samples were collected from both partners in the event of a positive test as a component of the Zambia-Emory HIV Research Project (ZEHRP). Dates for ART initiation were self-reported, and clinically-based estimated dates of infection (EDIs) were calculated with the appropriate formula of the three following: 1. midpoint of dates for the last antibody negative and first antibody positive test; 2. Fourteen days prior to the first p24 antigen positive, antibody negative test; 3. Ten days prior to the first viral load >1600 copies/mL, antibody negative test. All participants had antibody positive tests for their first HIV+ test, with the exception of participants Z1123M, Z1047M, and Z1808F. EDIs for participants Z1047M and Z1808F were estimated as in formula 2 above, and Z1123M EDI was calculated according to formula 3. For participant Z1047M, plasma from the first HIV+ test was not available for HIV sequence amplification, and therefore a sample from 10 days later on 24 Aug 2007 was used as the seroconversion sample. All other seroconversion samples were collected the day of the first HIV+ test.

### Ethics statement

Human subjects protocols for ZEHRP were approved by the University Teaching Hospital Ethics Committee in Lusaka, Zambia, while additional approval for sample or data use was granted by the Institutional Review Boards of Emory University, Simon Fraser University, and Providence Health Care/University of British Columbia. Written informed consent for sample collection was obtained for each volunteer upon enrollment in CVCT.

### Nucleic acid extraction and cDNA synthesis

Viral RNA in plasma samples was extracted using the QIAamp Viral RNA Mini Kit (QIAGEN) or E.Z.N.A Viral RNA Kit (Omega Bio-Tek) according to the manufacturer’s instructions. Briefly, 150 uL plasma was lysed with buffer and centrifuged through a silica column, which was then washed with appropriate buffers. RNA was eluted in >60 uL Buffer AVE (QIAGEN) or DEPC H_2_O (Omega). RNA served as template in cDNA synthesis reactions described below.

Eleven microliters of viral RNA were used in each 20 uL reverse-transcriptase reaction for cDNA synthesis utilizing SuperScript III or IV Reverse Transcriptase (Invitrogen) according to the manufacturer’s instructions, but with an extension time of up to one hour. SuperScript III protocols additionally included a 4°C pause following the one hour extension time for addition of 200 Units RT enzyme proceeding a second extension for two hours at 55°C. Both SuperScriot III and IV protocols included RNase H digestion of RNA-DNA heteroduplexes with 20 min incubations at 37°C. Oligo dT (5’-TTTTTTTTTTTTTTTTTT-3’) or 1.3’3’PlCb (5’-ACTACTTAGAGCACTCAAGGCAAGCTTTATTG-3’) primers were used as anchors in the reactions, and cDNA was directly used in PCR or frozen for subsequent use.

Nucleic acids were extracted from cells using the QIAamp DNA Blood Mini or Midi Kit (QIAGEN) according to the manufacturer’s instructions. Briefly, white cell pellets of total white blood cells in RNAlater were processed for lysis in QIAGEN Protease or Proteinase K and lysis buffer. Following addition of 100% ethanol, lysate was applied to a silica column and centrifuged, following by washing of the column. Samples were eluted in QIAGEN buffer AVE and used directly in PCR or frozen for subsequent use.

### PCR and amplicon purification

PCR for single genome amplification (SGA) of near full-length genomes (NFLGs) consisted of two rounds of PCR utilizing appropriate template for ≤40% positive reactions of approximately nine kilobases as visualized by gel electrophoresis. Each round of PCR consisted of 25 uL reactions with 0.5 Units Q5 Hot Start High-Fidelity Enzyme (NEB), 1x Q5 Reaction Buffer, 1x Q5 High GC Enhancer, 350 μM each dNTP, 500 nM each primer, plus template and nuclease-free H_2_O to reach 25 uL. PCR primers are described in Rousseau 2006 [47] for both first and second rounds, and first round primers are as follows: 1.U5Cc (5’-CCTTGAGTGCTCTAAGTAGTGTGTGCCCGTCTGT-3’, forward primer) and 1.3’3’PlCb (5’- ACTACTTAGAGCACTCAAGGCAAGCTTTATTG-3’, reverse primer). Second round PCR primers are as follows, with 1 uL of first round PCR product used as template in the second round PCR: 2.U5Cd (5’-AGTAGTGTGTGCCCGTCTGTTGTGTGACTC-3’, forward primer) and 2.3’3’plCb (5’-TAGAGCACTCAAGGCAAGCTTTATTGAGGCTTA-3’, reverse primer). Both first and second round PCR utilized the following program: 98°C for 30 sec, 35 cycles of 98°C for 10 sec and 72°C for 7:30 sec, 72°C for 10:00 min, and 4°C forever (end). Amplicons were purified with the Wizard SV Gel and PCR Clean-Up System (Promega) according to manufacturere’s instructions, eluting in H_2_O.

For amplification of *vpu*, *env*, and *nef* gene amplicons, two rounds of PCR were used as above for NFLG amplification. Reactions were 20 uL with first round primers: Vif1 KB (5’- GGGTTTATTACAGRGACAGCAGAG-3’, forward primer) and Ofm19 (5’-GCACTCAAGGCAAGCTTTATTGAGGCTTA-3’, reverse primer). First round PCR product (0.8 uL) was used a template for second round PCR with the following primers: EA1F KB (5’- GCTTAGGCATYTCMTATGGCAGGAAGAAG-3’, forward primer) and O1R (5’- AAAGCAGCTGCTTATATGCAGCWTC-3’, reverse primer). First round PCR program was as follows: 98°C for 45 sec, 30 cycles of 98°C for 15 sec, 60°C for 30 sec, and 72°C for 4:00 min, then 10 min at 72°C, and 4°C forever (end). Second round program was the same but for a 62°C annealing temperature and 3:00 min extension step. Amplicons were purified with the NucleoSpin Gel and PCR Clean-Up (Takara), eluting in Elution Buffer NE or H_2_O.

### Next-generation sequencing

All sequencing was performed with Pacific Biosciences SMRTbell sequencing on the RS II, with individual DNA libraries run on a single SMRT cell. Libraries were generated with 30–60 NFLG amplicons combined at eqiumolar concentrations and identified by nucleotide barcode following reamplification of first round PCR products with barcoded primers, or by barcoded adapter from the SMRTbell Barcoded Adapter Complete Prep Kit-96 (Pacific Biosciences). Libraries for *vpu*, *env*, and *nef* amplicons were generated with 80–100 amplicons per library and identified with barcoded adapters. Libraries were made according to manufacturer’s instruction, followed by appropriate size selection with the BluePippin (Sage Science). We are greatly appreciative of library size selection and quality control, as well as sample run of libraries on the RS II performed at the University of Delaware Sequencing and Genotyping Center.

Amplicon reads generated from the SMRT sequencing were analyzed with a unique algorithm to perform read phasing and error correction in generation of final sequences [33]. Libraries generated using Pacific Biosciences barcoded adapters were first analyzed with PacBio SMRT analysis software PB Barcode to separate reads by barcoded adapter prior to additional read phasing and error correction with the algorithm described in Dilernia et al. [33].

### Phylogenetic trees and reservoir variant dating

Maximum-likelihood phylogenetic trees for complete amplicons lacking frameshifting INDELs, APOBEC hypermutation, or other deleterious mutations were made with the PhyML plugin [48] of Geneious software v9.0.4 [49] using a general time reversible model with six nucleotide substitution categories and gamma distribution parameter with 100 bootstraps. APOBEC hypermutants were first removed from analysis with the LANL Hypermut v2.0 tool [50] with the appropriate transmitted/founder (TF) virus as the reference sequence, and all sequences of p<0.05 considered hypermutated. Trees were rooted on the appropriate TF virus sequence trimmed to the *vpu*, *env*, and *nef* gene amplicon. Patristic distances from the TF virus were extracted from the distance matrix. Trees were edited for visualization with MEGA v7.0.26 [51]. Statistics for patristic distance from phylogenetic trees were performed using Prism v8.3.0. Figures were made using Prism or, for Fig 1, JMP Pro 14 v14.2.

For all 12 participants with single-variant infections, we estimated the root date of their within-host plasma HIV-1 RNA sequences using established Bayesian methods. Briefly, within-host pre-therapy plasma HIV-1 *env* sequences were first screened for hypermutation (using Hypermut v2.0) and recombination (RDP v4.95 [52]) and any hypermutated or within-host recombinant sequences were removed. Sequences with ambiguous bases were also excluded, and identical sequences discarded but for one sequence from the earliest time point at which it was sampled. We ran two parallel 100,000,000 length chains sampling every 10,000 states in the software package BEAST v1.10.4 [53] for each participant. Posterior distributions for the root date were estimated using the unlinked SRD06 substitution model [54], the uncorrelated relaxed lognormal clock models [55], and the coalescent GMRF Bayesian skyride tree model [56, 57]. After discarding 10–30% of the initial run as burn-in, the chains from parallel runs were combined with LogCombiner v2.5.2 [58] and analyzed in Tracer v1.7.1 [59] to ensure convergence and verify that effective sample size values were >200 for all parameters.

Proviral variant integration dates were estimated as previously described [32] for the 12 participants infected by a single transmitted/founder virus. Briefly, *env* genes were trimmed from seroconversion and all other pre-therapy variants, as well as proviral variants from samples collected during treatment. Any sequences demonstrating hypermutation, ambiguous bases, or recombination were excluded from analysis, and only the earliest variant of duplicate sequences was kept for analysis. Maximum-likelihood trees were generated with RAxML v8.2.12 [60] and trees were rooted with root-to-tip regression (RTT) using the R package ape v5.3 [61] to maximize the correlation between the divergence from root and the sample collection date of the pre-therapy sequences. The pre-therapy variants were used to train a linear model of the divergence from root and the sample collection date. Finally, the integration dates date and confidence intervals of the proviral variants were estimated from this model.

## Supporting information

S1 Table Extended sampling details. (PDF)

S1 Fig Seroconversion sequences. (A) Maximum-likelihood phylogenetic tree of the near full-length HIV-1 genomes from all 13 study individuals at the seroconversion time point with color-coding of sequences from each participant. A single distinct clade and very short branch lengths within each participant viral population are indicative of the low sequence diversity, except in the case of participant Z1658F, where two clades are present. Sequences within each clade for Z1658F are low-diversity, consistent with infection being established by two transmitted/founder (TF) viruses. (B) Highlighter plot of the two viral populations in seroconversion sequences for Z1658F, with each TF virus as a master sequence. Polymorphisms matching TF virus A are shown in red, those matching TF virus B are in blue, and unique polymorphisms are in grey. Highlighter plot made with LANL Highlighter tool [62].
(PDF)

S2 Fig Maximum-likelihood (ML) trees for five participants with one sample available during treatment. (A) Z1094F (B) Z2006M (C) Z1808F (D) Z1044M and (E) Z1658F. Trees for participants with one sample during treatment not shown in Fig 3 are shown here. Participant Z1658F was infected with two transmitted/founder (TF) viruses, both included in grey in the ML tree, which is rooted on a Zambian subtype C consensus sequence (black square). All other trees are rooted on the respective TF virus (grey) identified from the seroconversion sample and depict all viral variants from one year post-infection (blue), the last ART-naïve sample (red), and during treatment (purple diamonds). Sequences from cells collected at the last ART-naïve time point are shown in open red diamonds, while all plasma variants are in filled circles.
(PDF)

S3 Fig Sequences during treatment are closer to transmitted/founder (TF) virus than last ART-naïve sequences. To compare distances across participants, each variant’s patristic distance from the TF virus or root is expressed as a proportion of the greatest patristic distance or branch length in a given participant’s maximum-likelihood tree. Means are shown in horizontal black bars. The proportional or scaled distances of sequences during treatment are significantly lower than sequences from either the cells or plasma at the last ART-naïve time point (Mann-Whitney tests).
(PDF)

S4 Fig Classifications of reservoir variants for each participant. Where sequences of the given era were not present and the percentage of the reservoir proviral population was therefore zero, the classification is omitted from the pie chart.
(PDF)

S5 Fig Proviral variant integration date estimates for each participant. All trees, linear models, and variant integration date estimates not shown in Fig 5 are provided here.
(PDF)
