# Supplementary material for: HIV-1 variants are archived throughout infection and persist in the reservoir
Source: PLoS Pathog. 2020 Jun 3;16(6):e1008378. doi: 10.1371/journal.ppat.1008378 (PMC7295247; doi:10.1371/journal.ppat.1008378)
Supplement: S1 Table — (PDF) [file ppat.1008378.s001.pdf]

Participant ID	Estimated Date of Infection (EDI)	First HIV+ Test Date	vRNA/ p24/ Ab at First HIV+ Test	SC Sample VL (copies/ mL)[a]	SC Sample # Seqs (AH)	EDI to 1 Yr Sample (Yrs)	1 Yr Sample VL (copies/ mL)	1 Yr Sample # Seqs (AH)	EDI to Last ART-naïve Sample (Yrs)	Last ART-naïve Sample VL (copies/ mL)	Last ART-naïve Sample # Seqs (AH) Plasma-Cells	ART Sample 1 VL (copies/ mL)	ART Sample 1 # Seqs (AH)	ART Sample 2 VL (copies/ mL)	ART Sample 2 # Seqs (AH)
Z1094F	21-May-2008	04-Jul-2008	+/NA/+	550,000	13 (0)	0.92	104,000	21 (0)	2.33	391,000	37 (0)-23 (2)	<50	21 (3)	NA	NA
Z1123M	02-Apr-2008	12-Apr-2008	+/-/-	37,068	8[b] (0)	1.01	40,500	23 (0)	2.82	110,259	30 (0)-22 (4)	<50	24 (6)	NA	NA
N133M	01-Jul-2007	16-Aug-2007	+/NA/+	180,220	22 (0)	0.68	100,001	23 (0)	2.77	186,000	25 (0)	<80	19 (1)	<50	18 (2)
Z1788F	26-Dec-2006	10-Feb-2007	+/NA/+	1,015,368	9 (0)	0.92	68,000	22 (0)	2.94	18,136	35 (0)-19 (5)	<50	22 (4)	<50	18 (1)
Z634F	08-Jul-2007	24-Jul-2007	+/NA/+	120,756	16 (0)	0.93	64,600	34 (0)	2.98	58,652	21 (0)	<50	21 (2)	<50	11 (3)
Z2006M	26-Apr-2009	13-May-2009	+/NA/+	27,200	9 (2)	0.93	10,900	26 (1)	3.22	109,070	23 (4)	<50	14 (7)	NA	NA
Z1047M	31-Jul-2007	14-Aug-2007[c]	+/+/-	17,300	5 (2)	0.92	538,000	20 (1)	3.53	305,564	36 (0)-21 (3)	<50	21 (3)	NA	NA
Z1165M	26-Jan-2006	16-Mar-2006	+/NA/+	>750,000	11 (0)	0.96	122,000	40 (0)	3.72	24,864	24 (0)-23 (0)	<50	17 (5)	<50	14 (3)
Z1658F	24-Apr-2006	08-Jun-2006	+/NA/+	219,111	16 (0)	0.93	44,500	17 (9)	4.62	34,370	28 (2)-20 (4)	<50	19 (9)	NA	NA
Z1808F	10-Jan-2008	24-Jan-2008	+/+/-	1,742,240	10 (0)	0.92	126,248	32 (0)	4.43	57,460	27 (14)	<50	16 (11)	NA	NA
Z1124F	02-Apr-2006	18-May-2006	+/NA/+	333,392	10 (0)	0.92	15,100	26 (0)	4.81	1,072	23 (0)	<50	15 (4)	NA	NA
Z1044M	26-Jan-2006	25-Mar-2006	+/NA/+	119,075	11 (0)	1.15	6,020	21 (0)	5.01	5,287	22 (0)	<50	19 (2)	NA	NA
Z326M	04-Jan-2007	17-Feb-2007	+/NA/+	44,800	9 (0)	1.39	32,700	31 (1)	6.22	9,100	23 (2)	<50	17 (0)	NA	NA
**MEAN**	**NA**	**NA**	**NA**	**396,656**	**11 (0)**	**0.97**	**97,890**	**26 (1)**	**3.80**	**100,833**	**27 (2)-22 (3)**	**<50**	**19 (4)**	**<50**	**15 (2)**
**MEDIAN**	**NA**	**NA**	**NA**	**180,220**	**10 (0)**	**0.93**	**64,600**	**23 (0)**	**3.53**	**57,460**	**25 (0)-23 (3)**	**<50**	**19 (4)**	**<50**	**16 (3)**

SC=Seroconversion
Seqs=Sequences
AH=additional APOBEC Hypermutant sequences excluded from analysis
[a]VL within seven days of sample collection
[b]Two additional sequences amplified for Z1123M from sample collected 15 May 2008 for 10 total sequences
[c]Z1047M seroconversion sample collected 10 days after first HIV+ test
