# Supplementary figures and images for: HIV-1 variants are archived throughout infection and persist in the reservoir
Source: PLoS Pathog. 2020 Jun 3;16(6):e1008378. doi: 10.1371/journal.ppat.1008378 (PMC7295247; doi:10.1371/journal.ppat.1008378)

**A**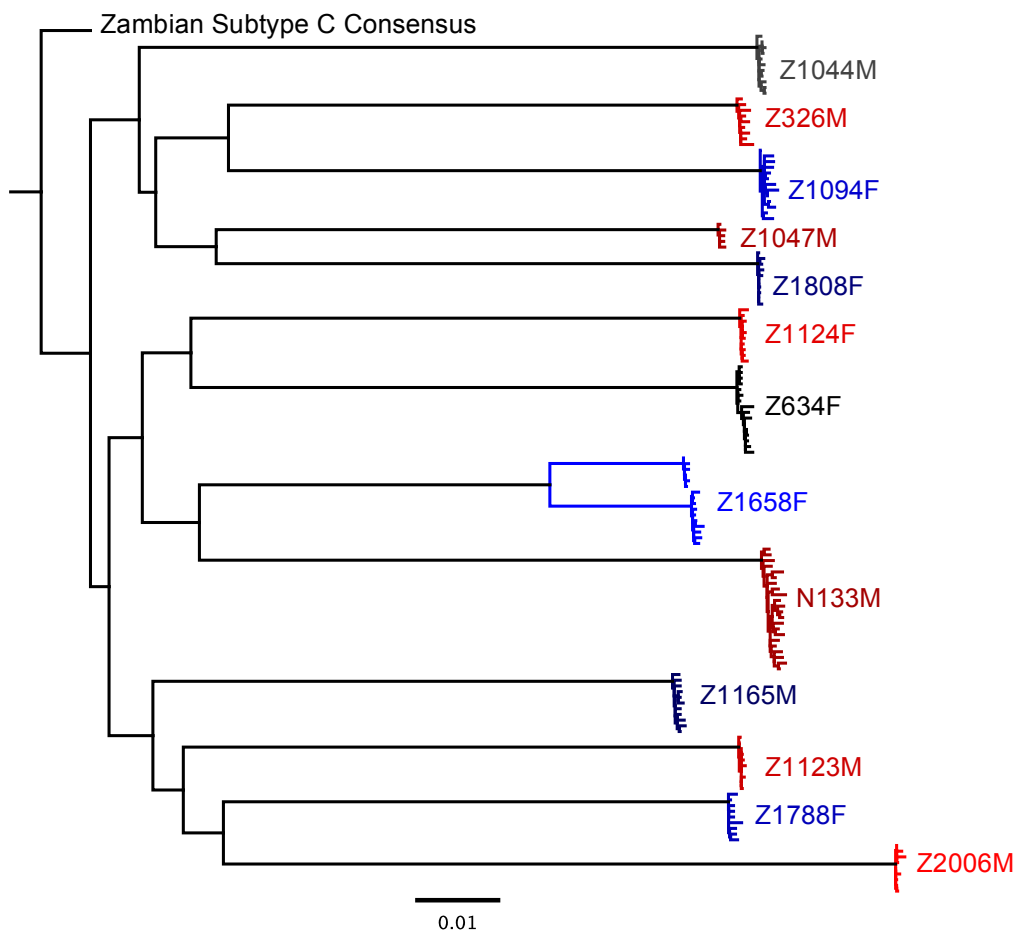**B**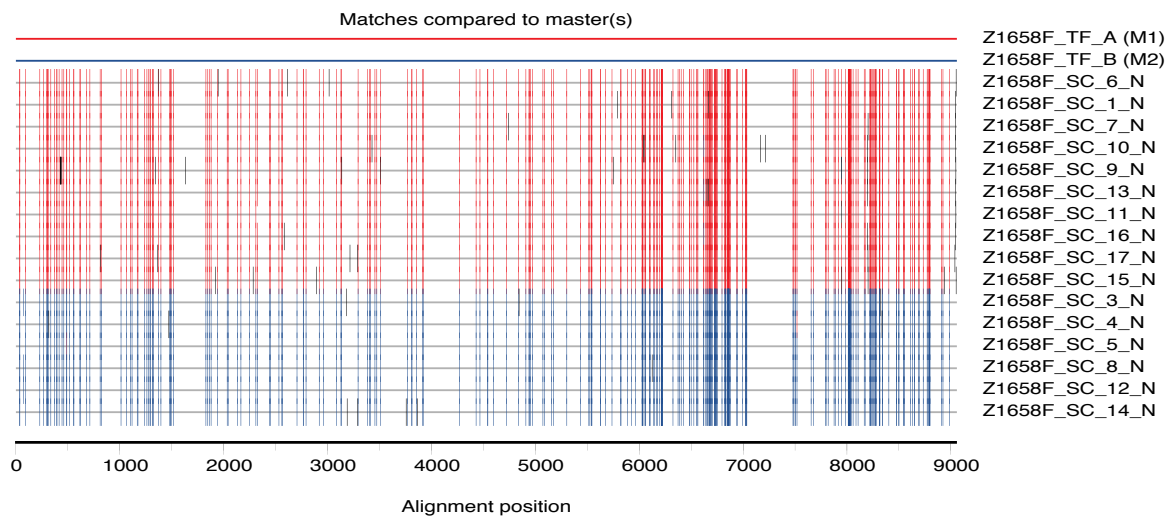

Supplement: S1 Fig — (A) Maximum-likelihood phylogenetic tree of the near full-length HIV-1 genomes from all 13 study individuals at the seroconversion time point with color-coding of sequences from each participant. A single distinct clade and very short branch lengths within each participant viral population are indicative of the low sequence diversity, except in the case of participant Z1658F, where two clades are present. Sequences within each clade for Z1658F are low-diversity, consistent with infection being established by two transmitted/founder (TF) viruses. (B) Highlighter plot of the two viral populations in seroconversion sequences for Z1658F, with each TF virus as a master sequence. Polymorphisms matching TF virus A are shown in red, those matching TF virus B are in blue, and unique polymorphisms are in grey. Highlighter plot made with LANL Highlighter tool [62]. (PDF) [file ppat.1008378.s002.pdf]

A

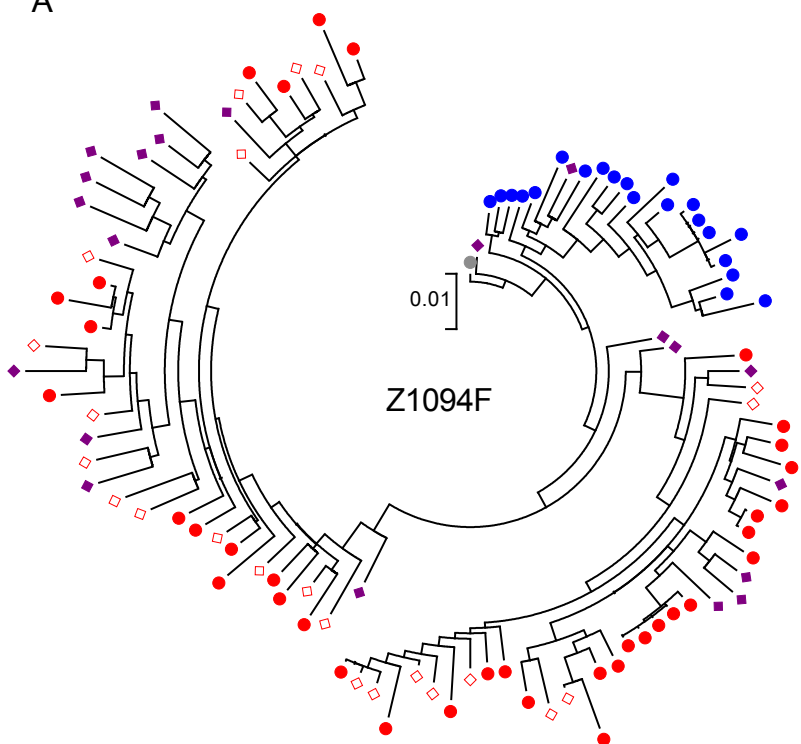

B

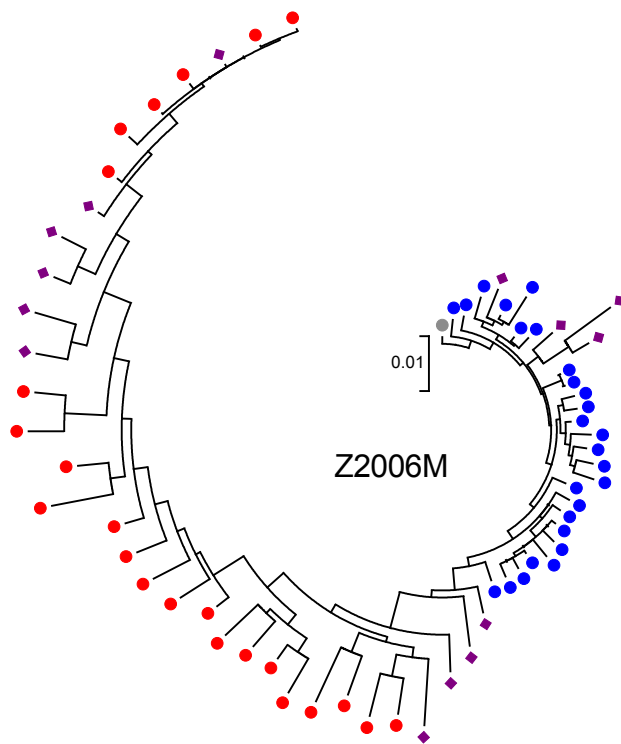

C

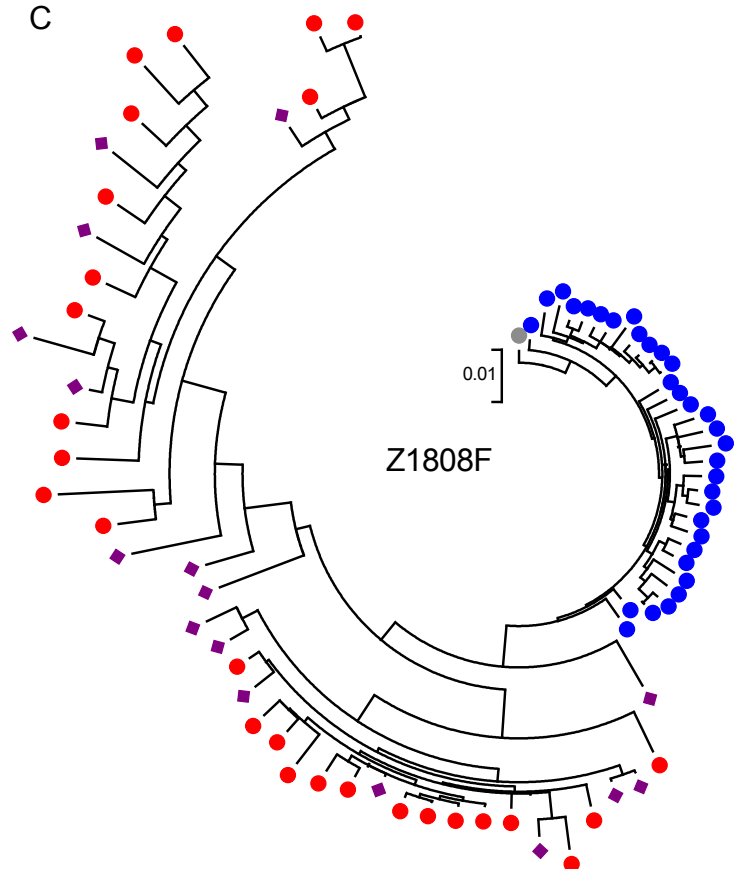

D

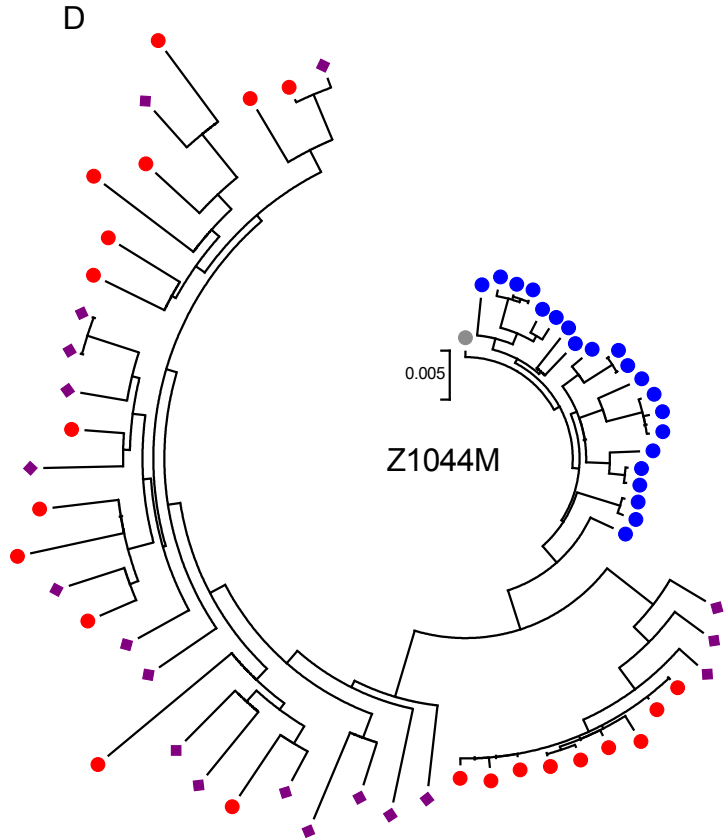

E

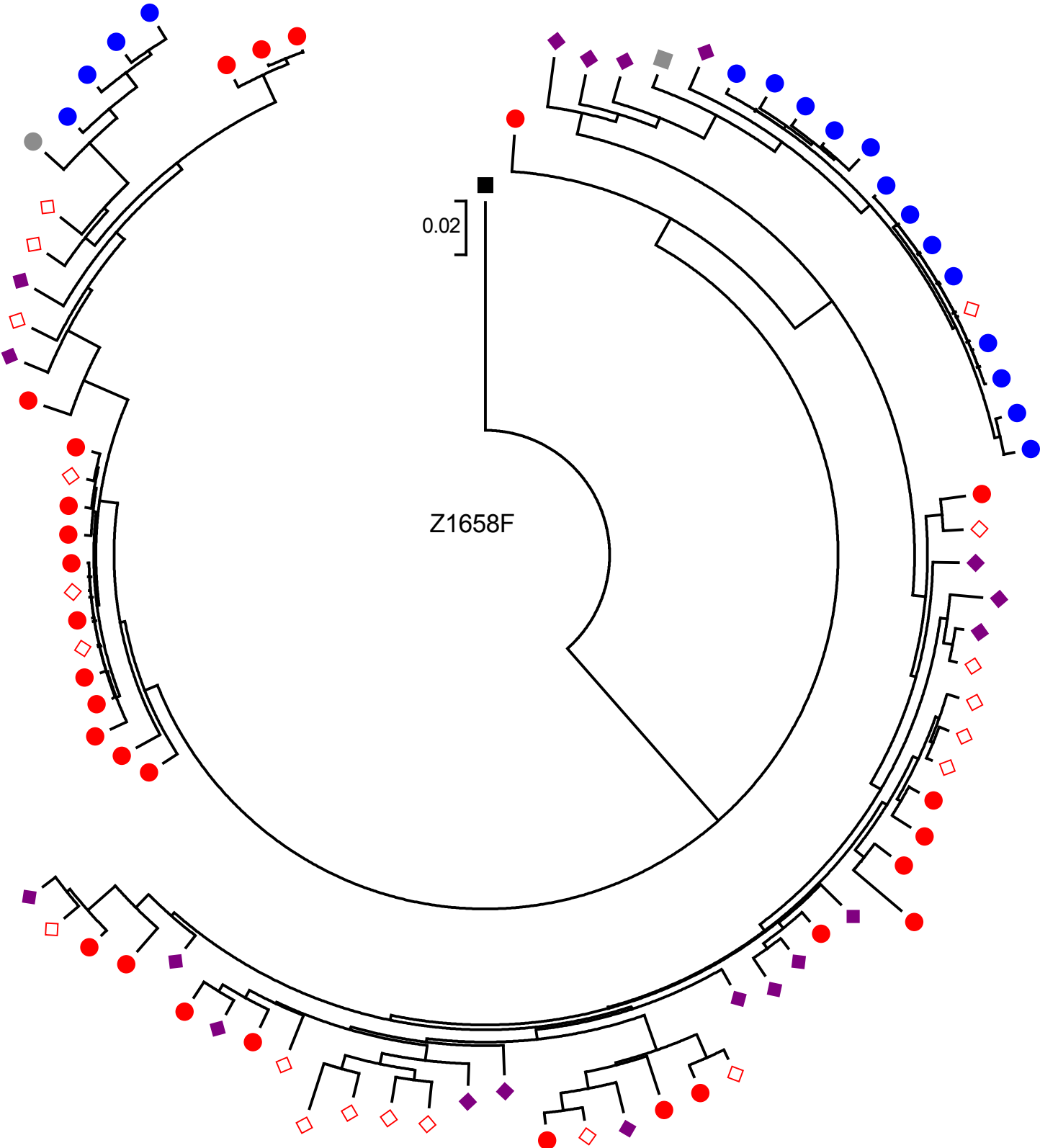

Supplement: S2 Fig — (A) Z1094F (B) Z2006M (C) Z1808F (D) Z1044M and (E) Z1658F. Trees for participants with one sample during treatment not shown in Fig 3 are shown here. Participant Z1658F was infected with two transmitted/founder (TF) viruses, both included in grey in the ML tree, which is rooted on a Zambian subtype C consensus sequence (black square). All other trees are rooted on the respective TF virus (grey) identified from the seroconversion sample and depict all viral variants from one year post-infection (blue), the last ART-naïve sample (red), and during treatment (purple diamonds). Sequences from cells collected at the last ART-naïve time point are shown in open red diamonds, while all plasma variants are in filled circles. (PDF) [file ppat.1008378.s003.pdf]

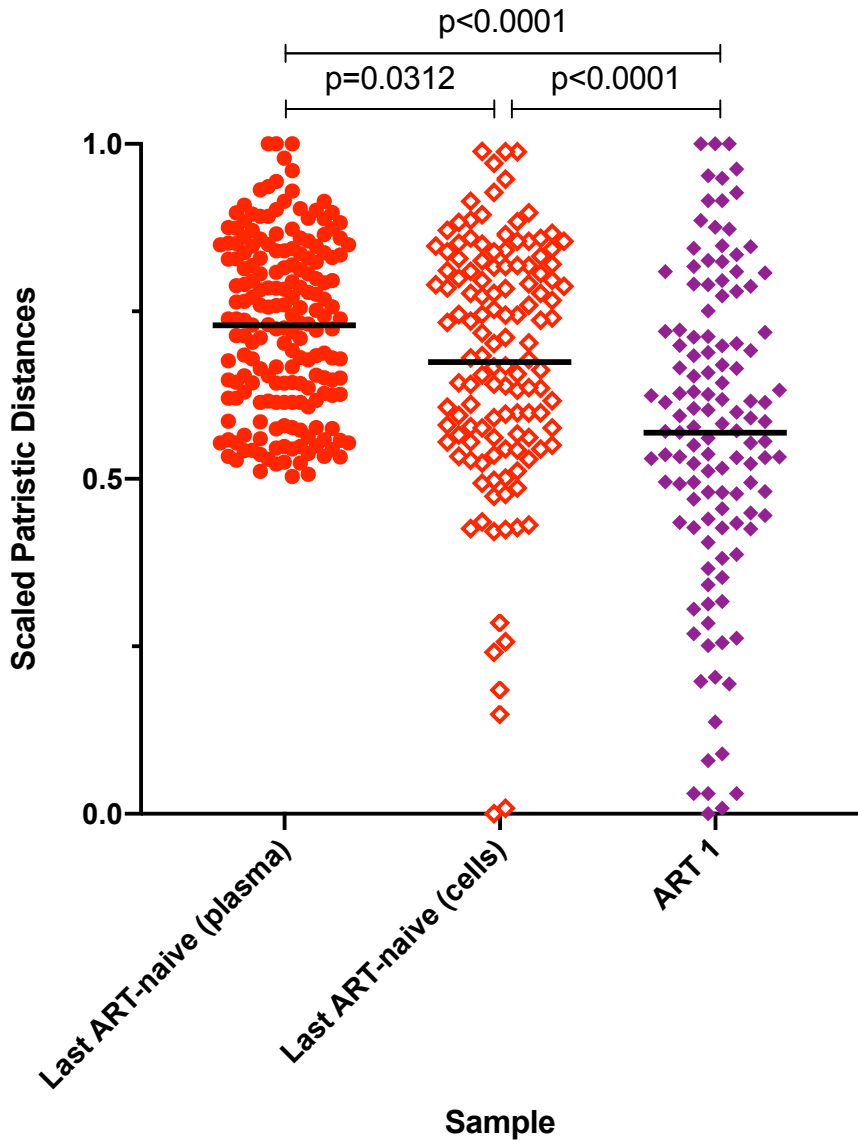

Supplement: S3 Fig — To compare distances across participants, each variant’s patristic distance from the TF virus or root is expressed as a proportion of the greatest patristic distance or branch length in a given participant’s maximum-likelihood tree. Means are shown in horizontal black bars. The proportional or scaled distances of sequences during treatment are significantly lower than sequences from either the cells or plasma at the last ART-naïve time point (Mann-Whitney tests). (PDF) [file ppat.1008378.s004.pdf]

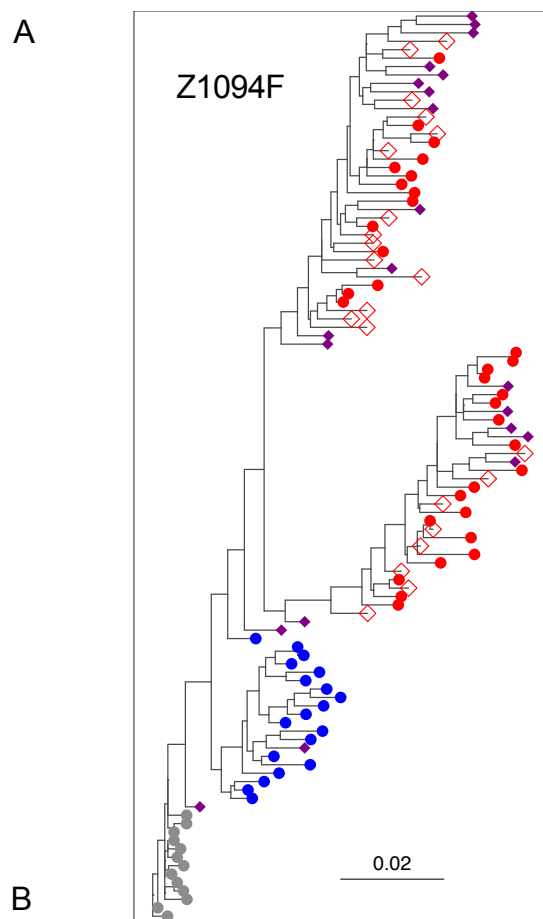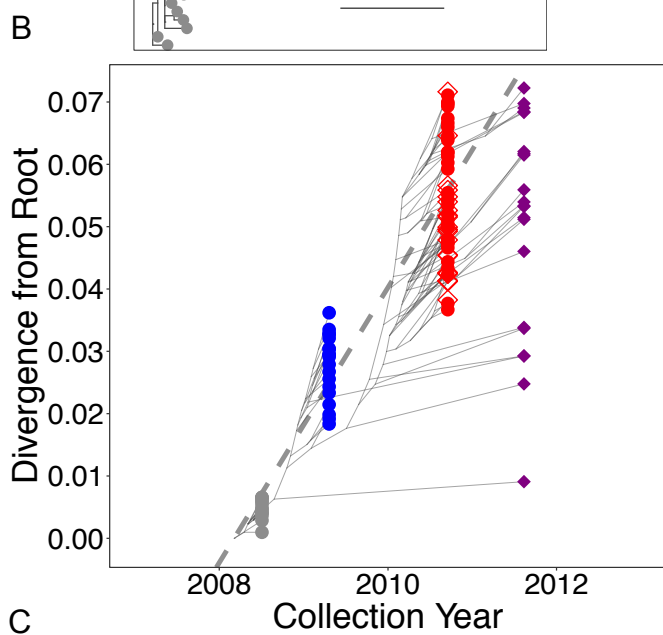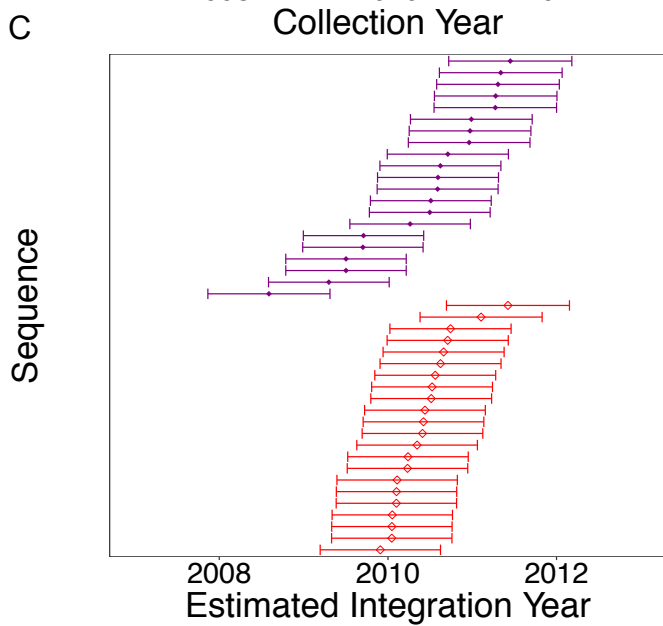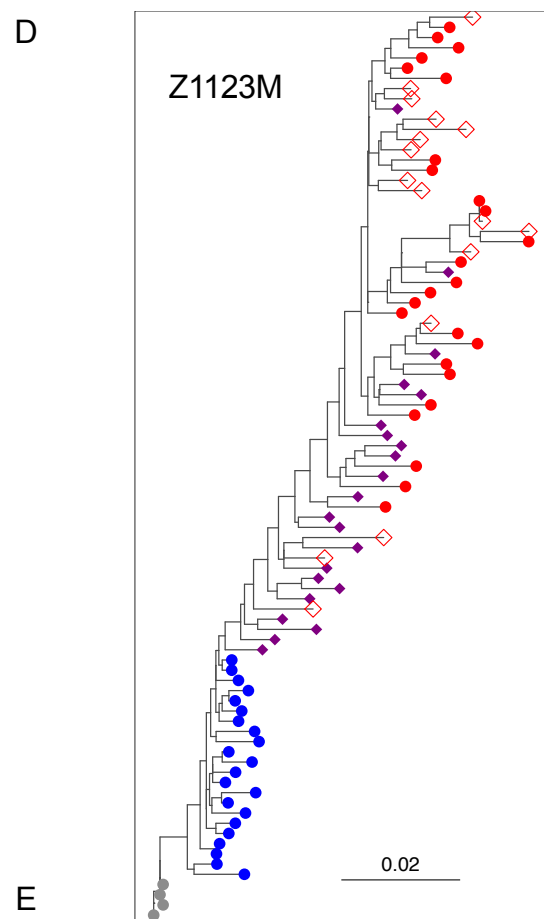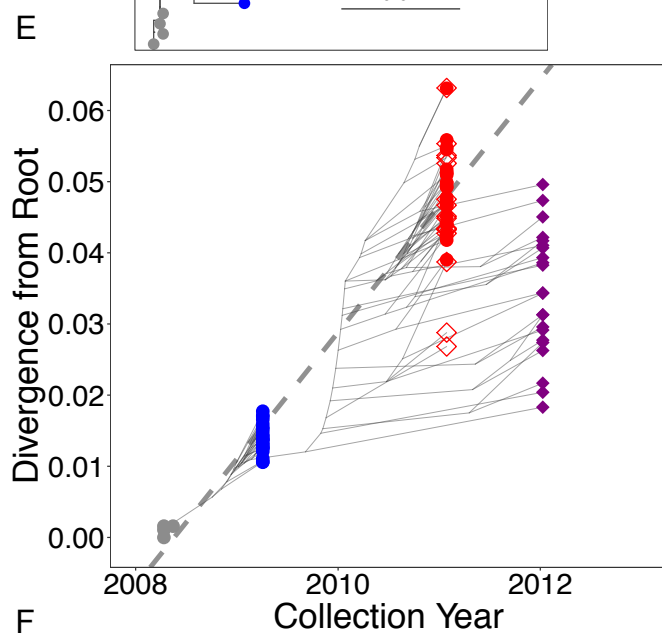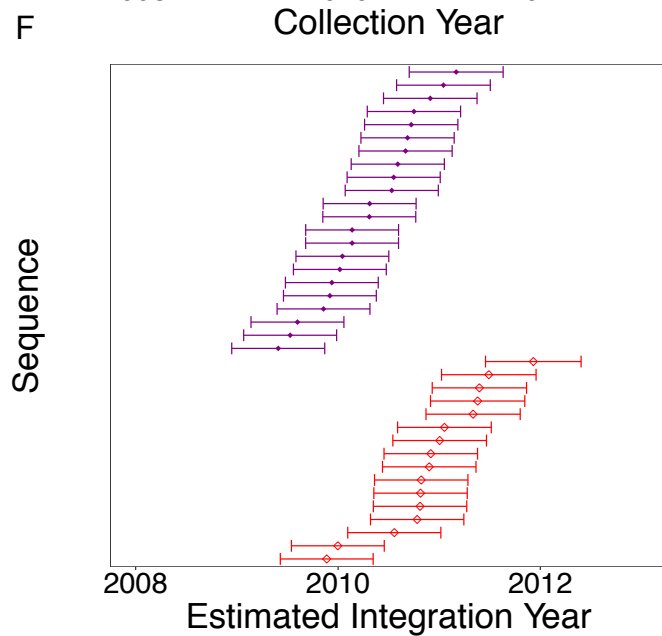

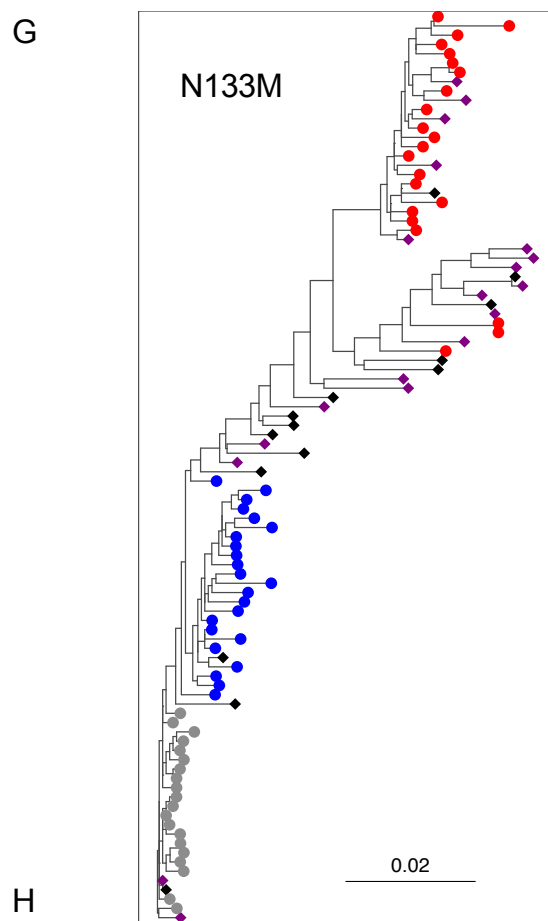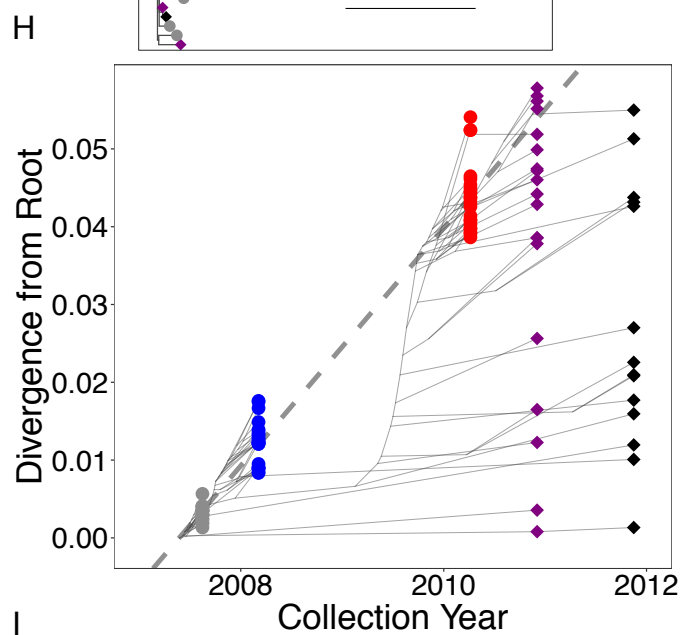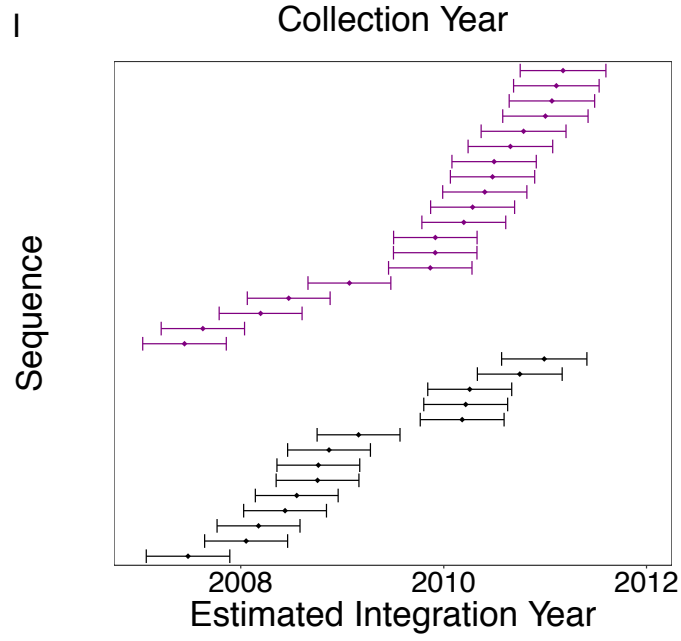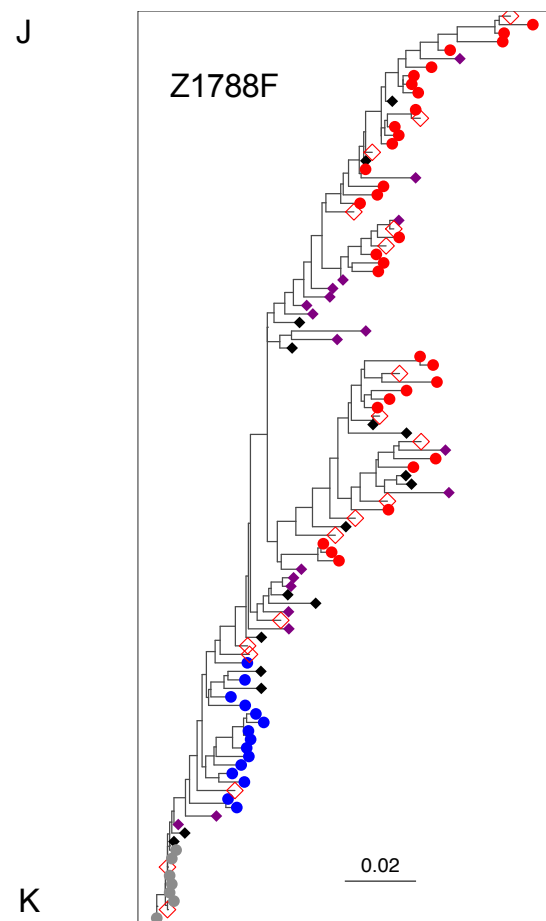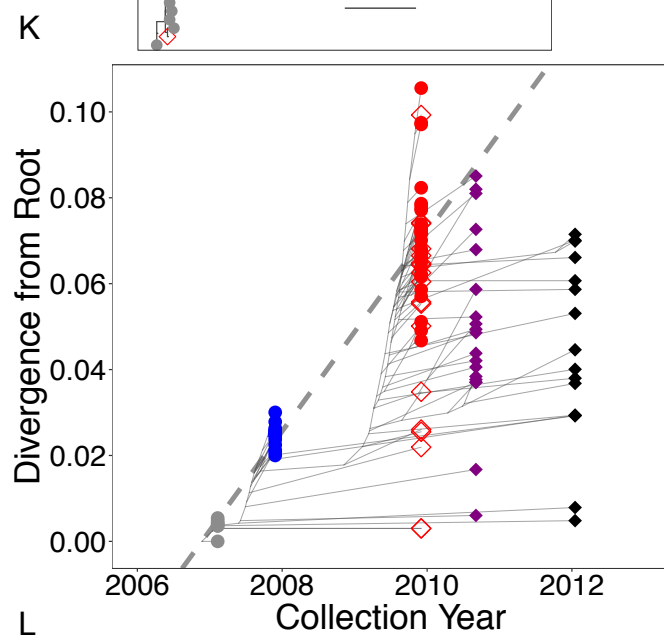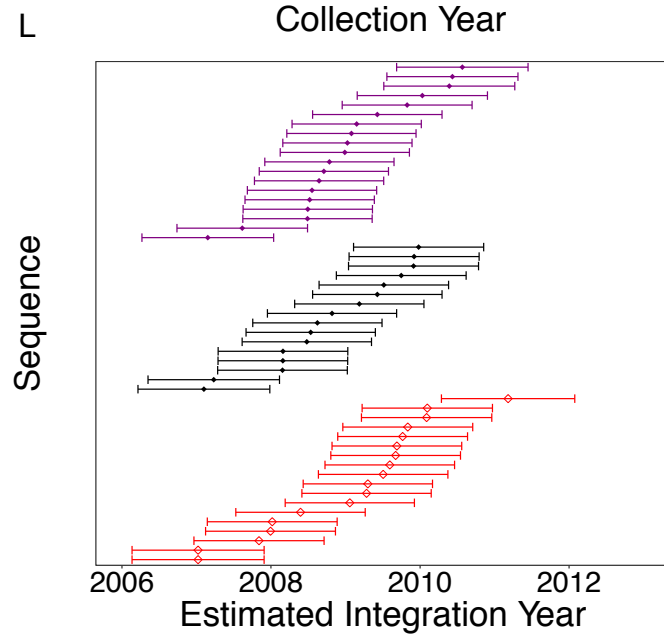

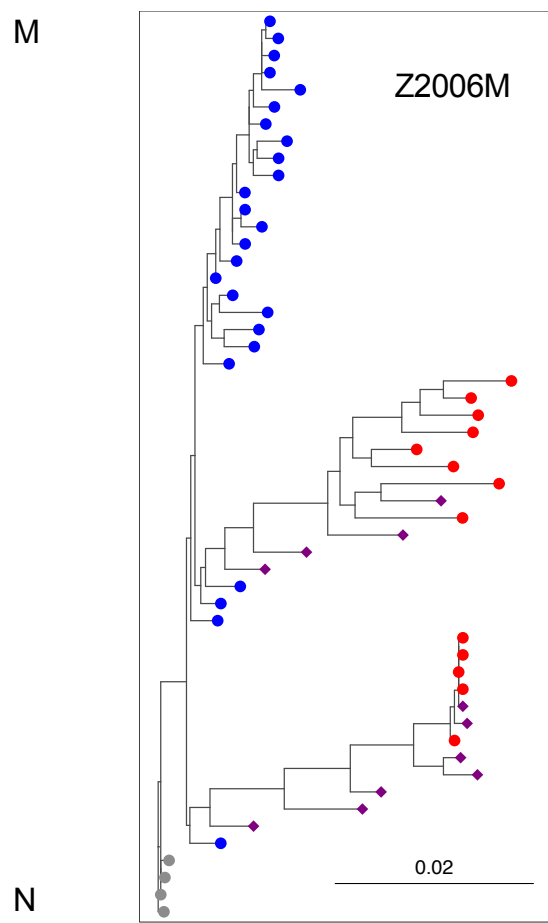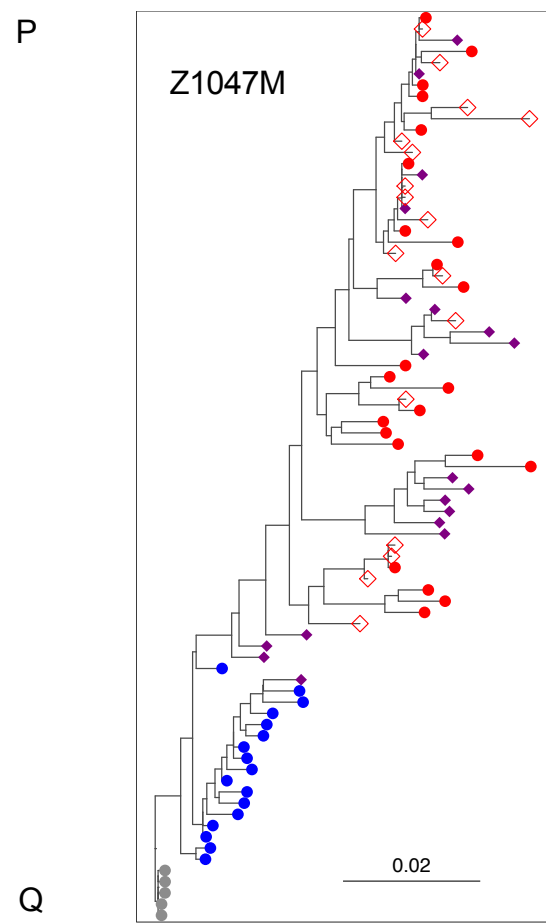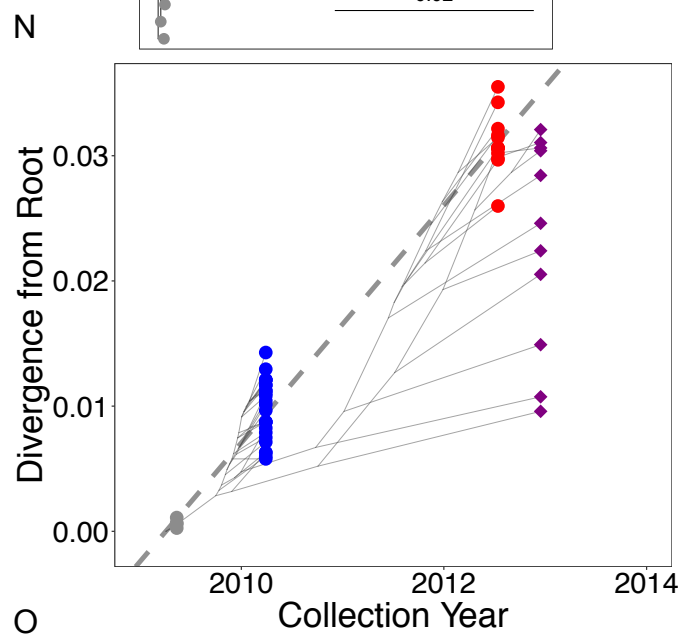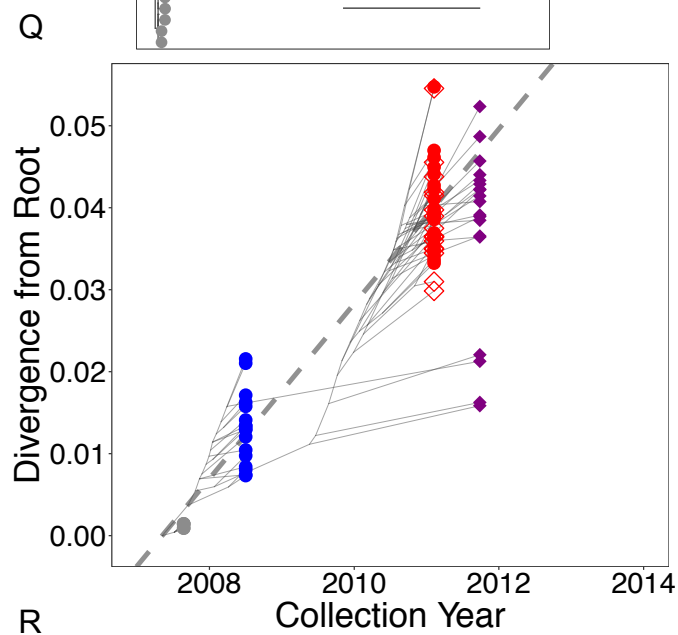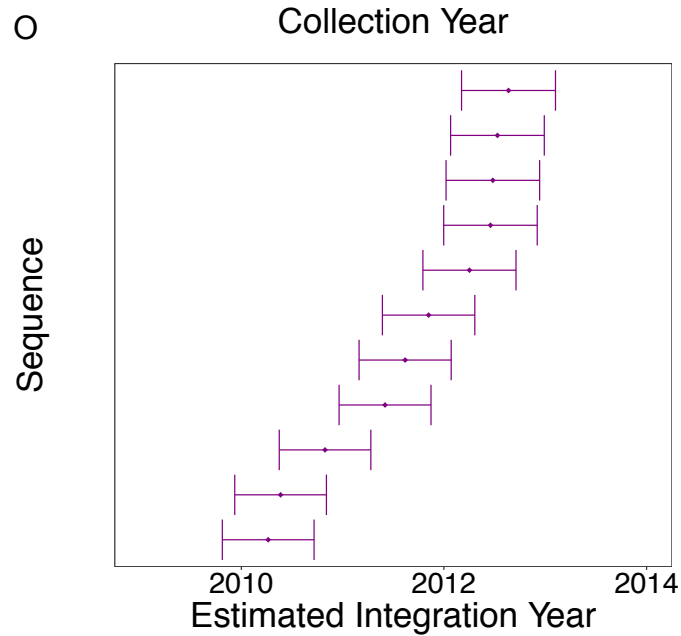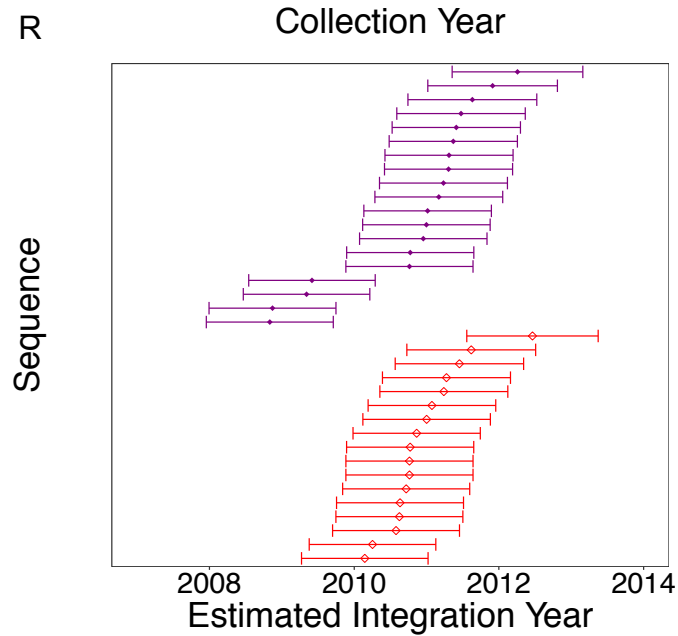

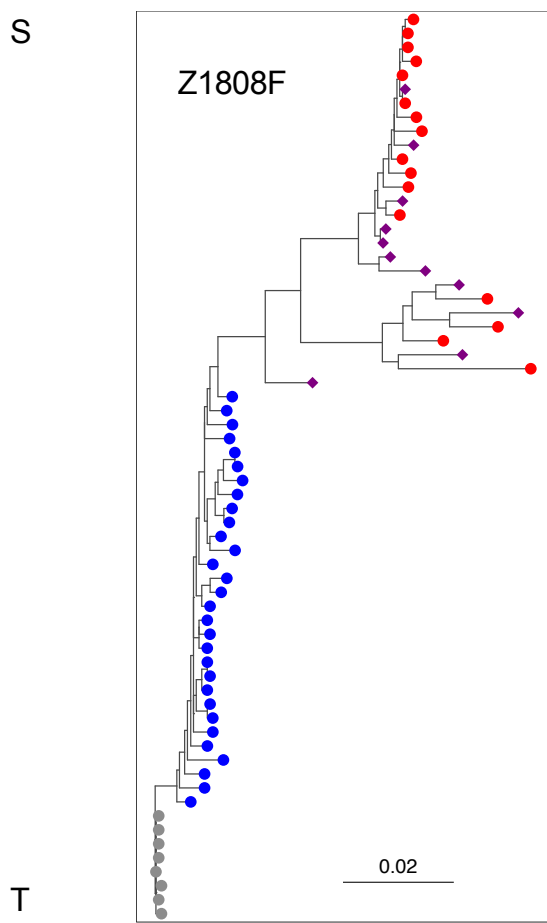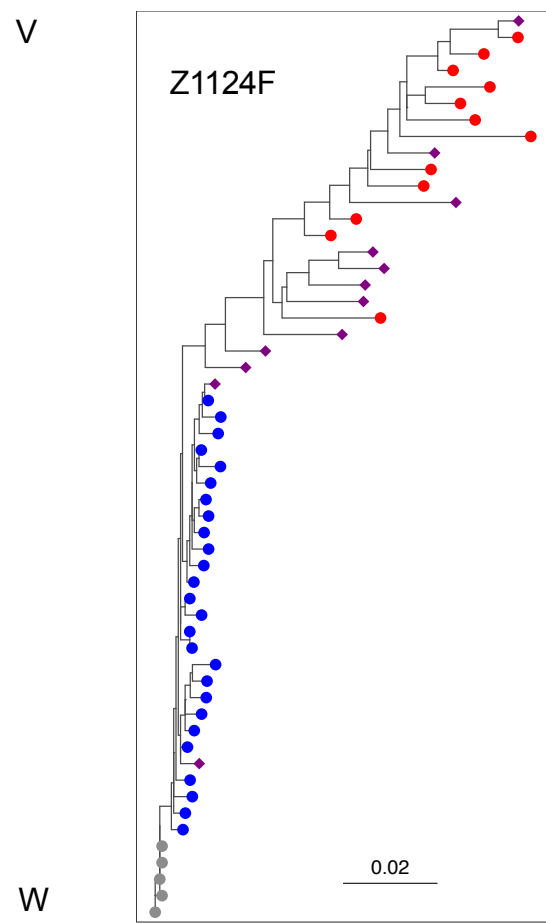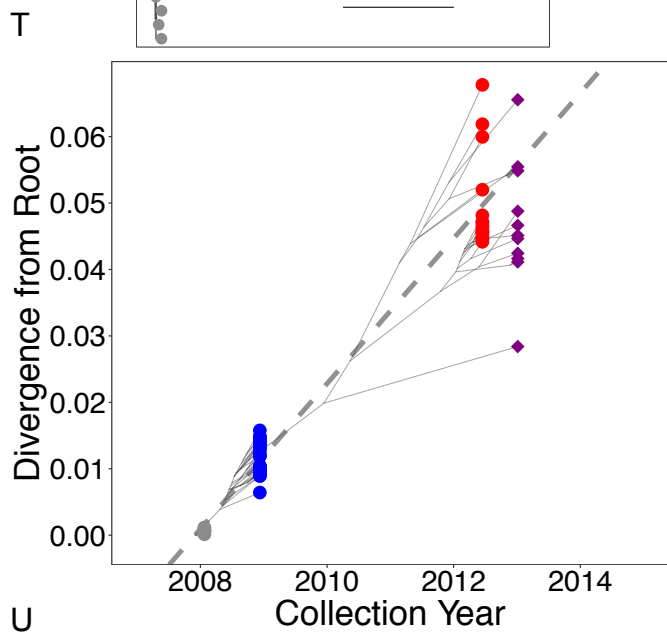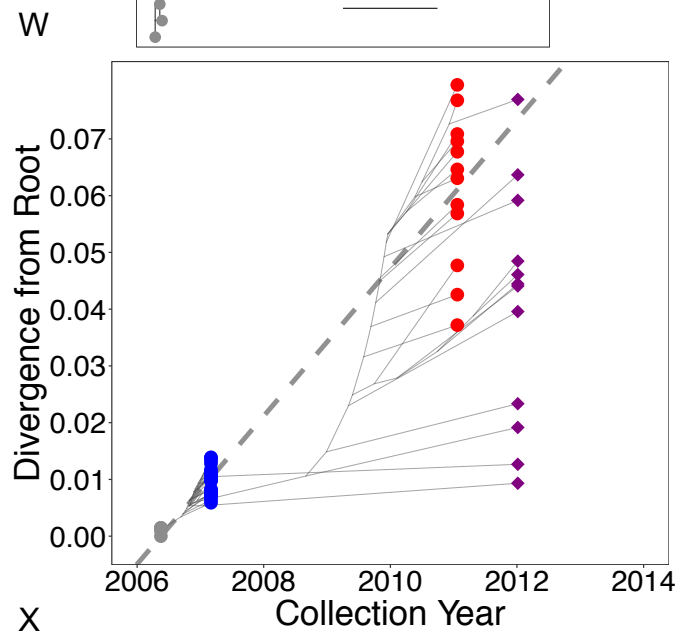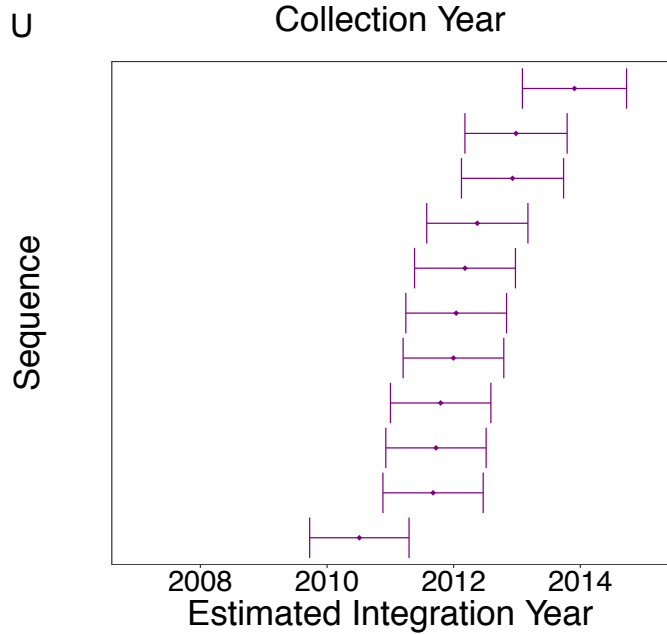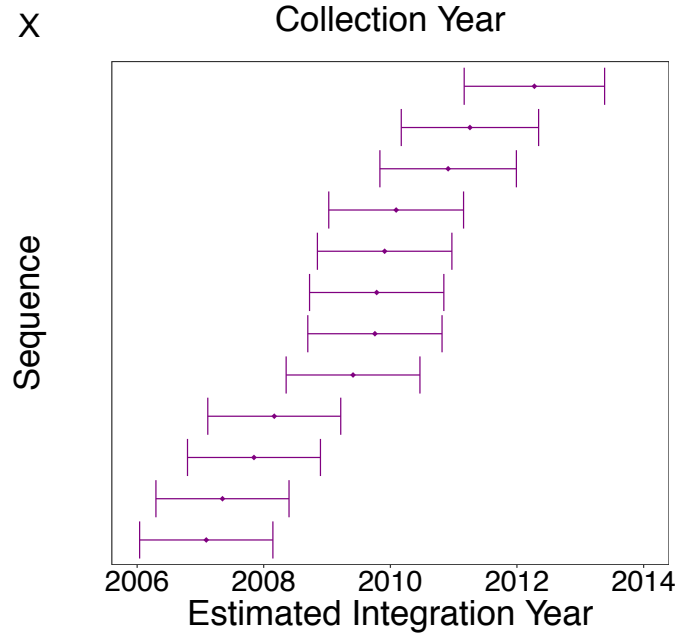

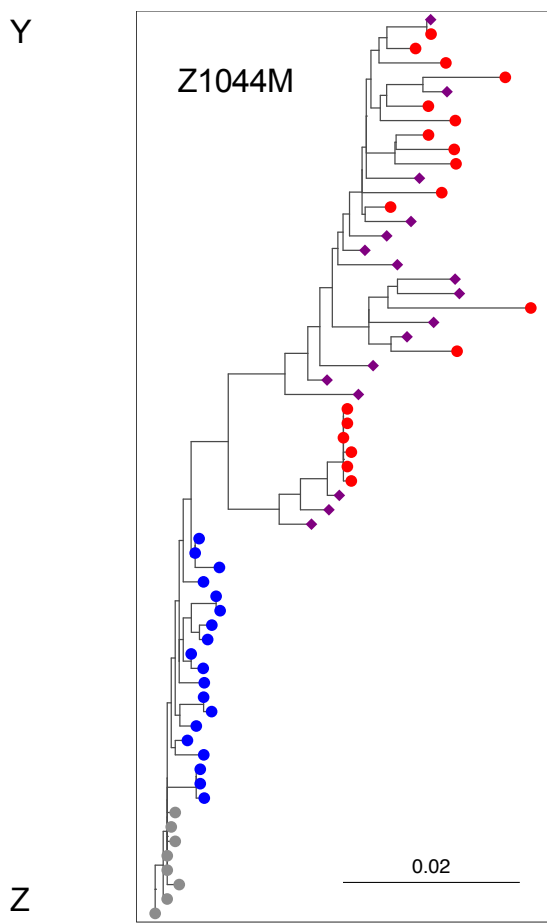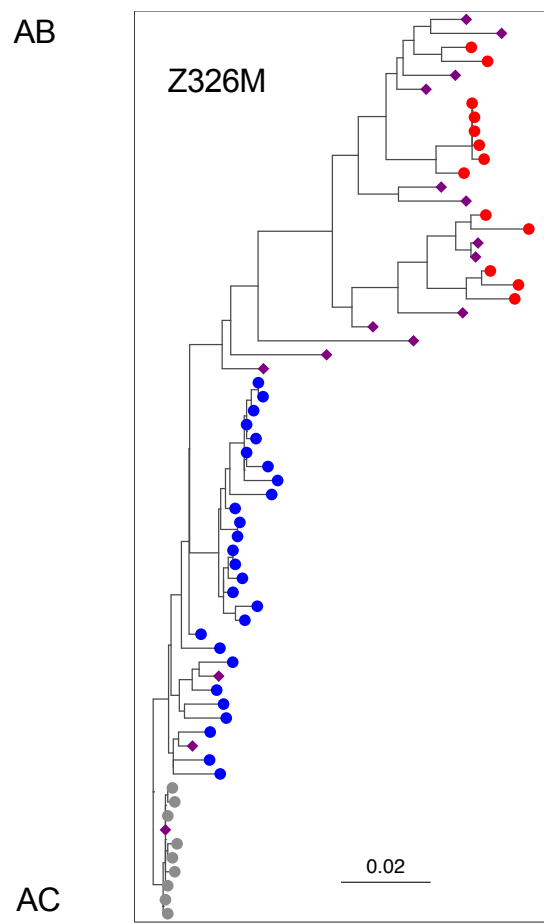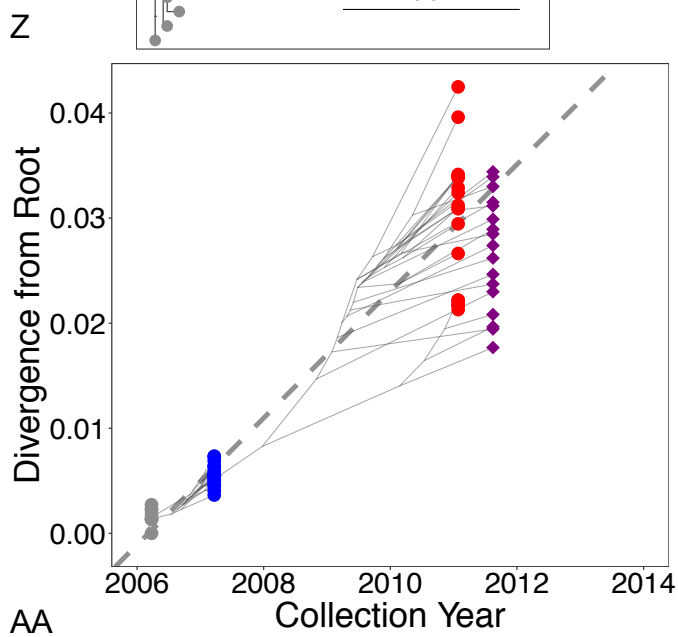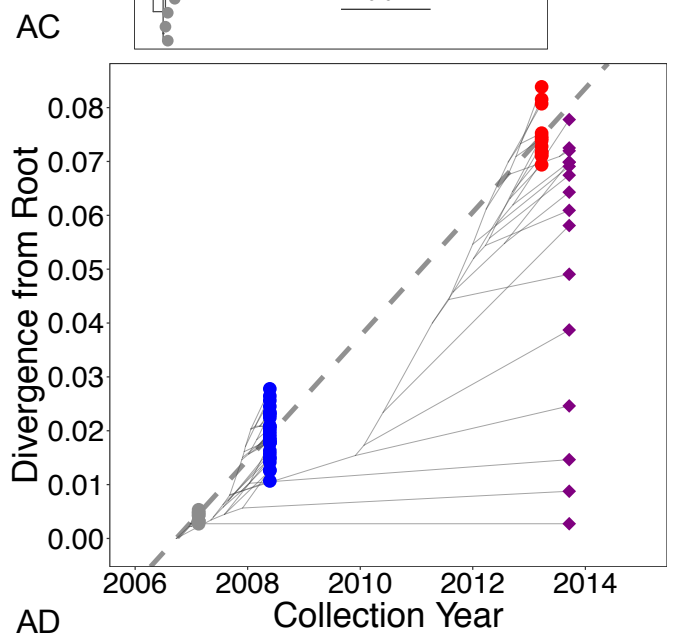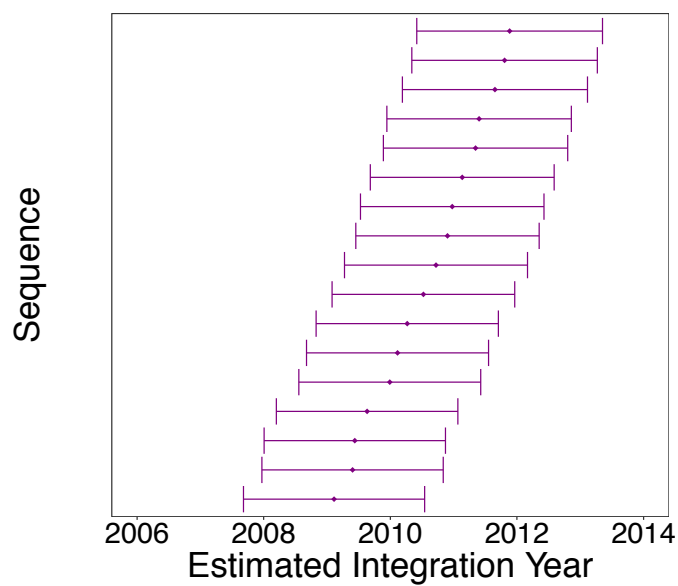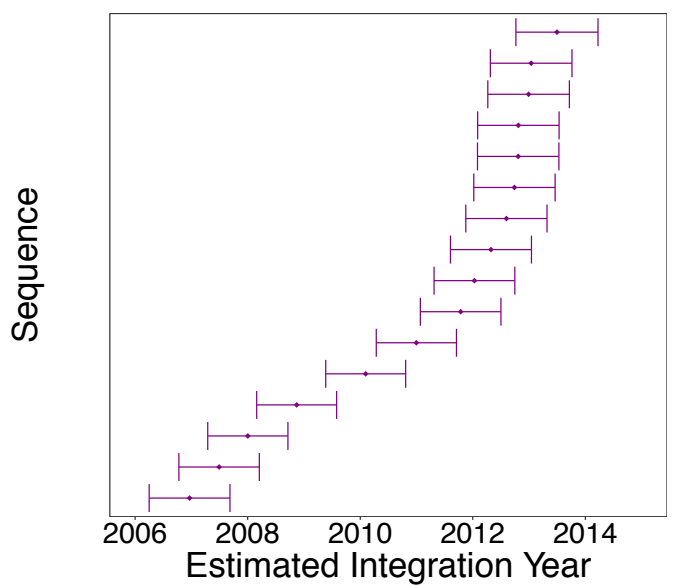

Supplement: S5 Fig — All trees, linear models, and variant integration date estimates not shown in Fig 5 are provided here. (PDF) [file ppat.1008378.s006.pdf]
