# Supplementary material for: HIV-1 variants are archived throughout infection and persist in the reservoir
Source: PLoS Pathog. 2020 Jun 3;16(6):e1008378. doi: 10.1371/journal.ppat.1008378 (PMC7295247; doi:10.1371/journal.ppat.1008378)
Supplement: S4 Fig — Where sequences of the given era were not present and the percentage of the reservoir proviral population was therefore zero, the classification is omitted from the pie chart. (PDF) [file ppat.1008378.s005.pdf]

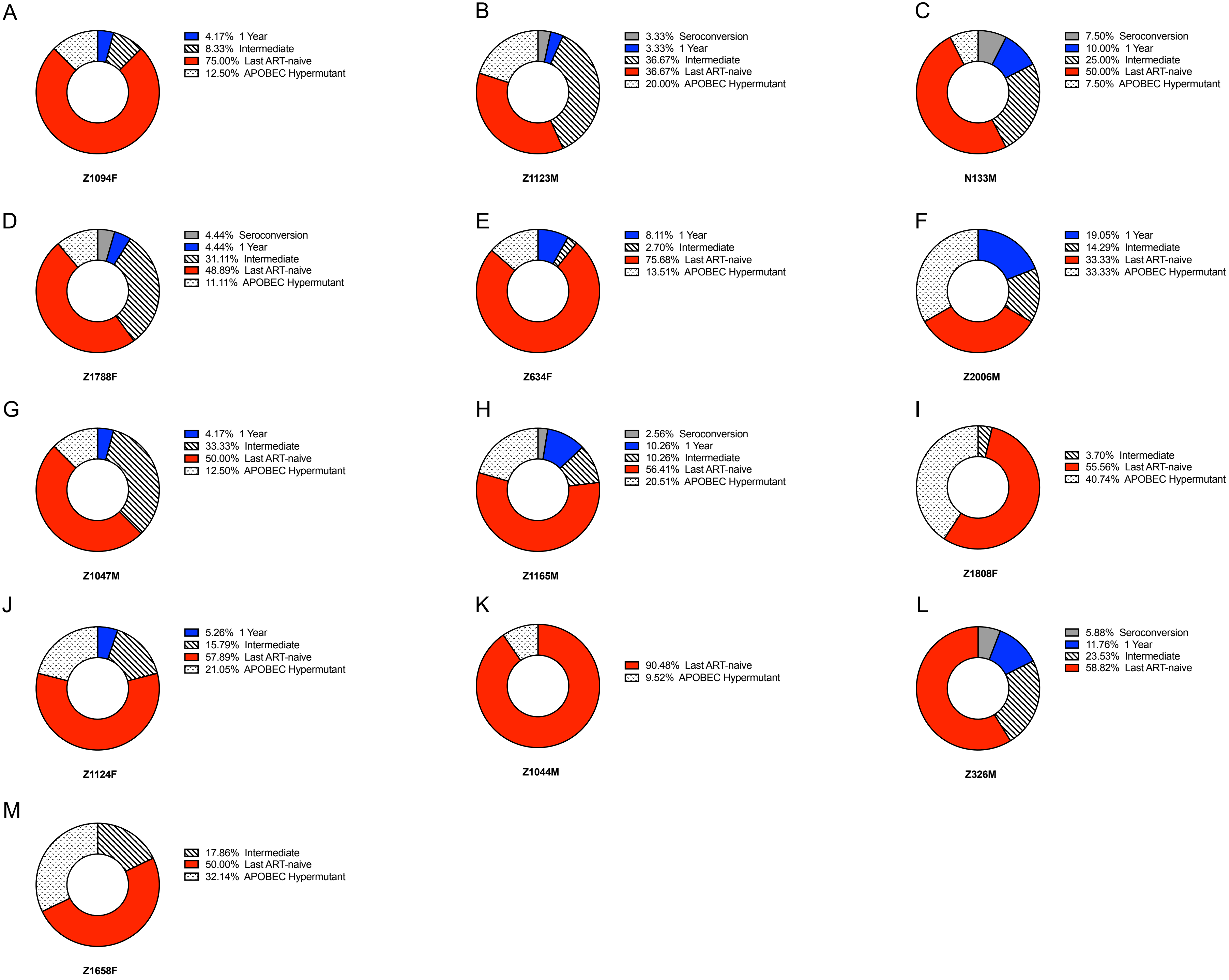
A
4.17% 1 Year
8.33% Intermediate
75.00% Last ART-naive
12.50% APOBEC Hypermutant
Z1094F
B
3.33% Seroconversion
3.33% 1 Year
36.67% Intermediate
36.67% Last ART-naive
20.00% APOBEC Hypermutant
Z1123M
C
7.50% Seroconversion
10.00% 1 Year
25.00% Intermediate
50.00% Last ART-naive
7.50% APOBEC Hypermutant
N133M
D
4.44% Seroconversion
4.44% 1 Year
31.11% Intermediate
48.89% Last ART-naive
11.11% APOBEC Hypermutant
Z1788F
E
8.11% 1 Year
2.70% Intermediate
75.68% Last ART-naive
13.51% APOBEC Hypermutant
Z634F
F
19.05% 1 Year
14.29% Intermediate
33.33% Last ART-naive
33.33% APOBEC Hypermutant
Z2006M
G
4.17% 1 Year
33.33% Intermediate
50.00% Last ART-naive
12.50% APOBEC Hypermutant
Z1047M
H
2.56% Seroconversion
10.26% 1 Year
10.26% Intermediate
56.41% Last ART-naive
20.51% APOBEC Hypermutant
Z1165M
I
3.70% Intermediate
55.56% Last ART-naive
40.74% APOBEC Hypermutant
Z1808F
J
5.26% 1 Year
15.79% Intermediate
57.89% Last ART-naive
21.05% APOBEC Hypermutant
Z1124F
K
90.48% Last ART-naive
9.52% APOBEC Hypermutant
Z1044M
L
5.88% Seroconversion
11.76% 1 Year
23.53% Intermediate
58.82% Last ART-naive
Z326M
M
17.86% Intermediate
50.00% Last ART-naive
32.14% APOBEC Hypermutant
Z1658F
